# Obesity: Prevalence, causes, consequences, management, preventive strategies and future research directions

**DOI:** 10.1016/j.metop.2025.100375

**Published:** 2025-06-14

**Authors:** Sirwan Khalid Ahmed, Ribwar Arsalan Mohammed

**Affiliations:** College of Nursing, University of Raparin, Rania, Sulaymaniyah, Kurdistan Region, 46012, Iraq

**Keywords:** Obesity, Overweight, Non-communicable diseases, Epidemiology, Global burden, Public health, Prevention strategies

## Abstract

Obesity has emerged as one of the most pressing global public health challenges of the 21st century. Obesity has reached epidemic proportions worldwide, with over 1 billion people classified as obese in 2022, representing 13 % of the global population. Since 1975, obesity rates have tripled, and projections indicate that by 2035, around 1.9 billion adults—approximately 25 % of the world's population—will be affected. Looking further ahead to 2050, it is estimated that 3.80 billion adults, representing more than half of the anticipated global adult population, will be living with overweight or obesity. The increasing burden of obesity is associated with an alarming rise in non-communicable diseases, including type 2 diabetes, cardiovascular diseases, and multiple cancers, collectively contributing to over 5 million deaths annually. Obesity is driven by complex interactions between genetic, behavioral, environmental, and socioeconomic factors, with rapid urbanization and globalization accelerating the consumption of high-calorie diets and sedentary lifestyles. While historically prevalent in high-income nations, obesity rates are now rising most rapidly in low- and middle-income countries (LMICs), with over 70 % of obese individuals living in developing nations. The economic costs of obesity are staggering, with projections estimating a global financial burden of $4.32 trillion per year by 2035, equivalent to 3 % of the global GDP. This article explores the epidemiology, determinants, health implications, and policy responses to obesity, emphasizing the urgent need for multisectoral strategies to mitigate its impact. Public health initiatives, taxation on sugar-sweetened beverages, improved food regulations, and increased physical activity promotion are essential components of evidence-based interventions. Addressing the obesity crisis requires global cooperation to implement sustainable, long-term strategies targeting both prevention and treatment.

## 1 Introduction

Obesity has emerged as one of the most pressing global public health challenges of the 21st century. In 2022, more than 1 billion people worldwide were living with obesity (body mass index ≥30) – roughly one in eight adults – and about 43 % of adults were classified as overweight or obese [1]. The prevalence of adult obesity has more than doubled since 1990, and childhood obesity rates have increased over four-fold in that time [1]. Such dramatic trends have led experts to characterize obesity as a global epidemic or even “pandemic” in scope [2]. In other words, excess adiposity is no longer a concern of only a few countries; it now affects populations in virtually every region on the planet.

Notably, the obesity epidemic is a truly worldwide phenomenon, transcending socioeconomic and geographic boundaries [[3], [4], [5], [6], [7]]. Once associated primarily with affluent Western societies, obesity now burdens low- and middle-income countries at accelerating rates [[8], [9], [10]]. Indeed, more people are obese than underweight in every global region except parts of sub-Saharan Africa and South-East Asia [1,[11], [12], [13]] Many nations face a double burden of malnutrition, where high obesity prevalence coexists with persistent undernutrition and micronutrient deficiencies [1,[14], [15], [16], [17], [18], [19], [20], [21], [22], [23], [24], [25], [26], [27]] This dual challenge is evident as some of the fastest growth in obesity is seen in urbanizing, lower-income settings that still struggle with food insecurity [[28], [29], [30], [31], [32], [33], [34], [35], [36], [37], [38], [39], [40], [41]]. The future trajectory is equally alarming: if current trends persist, over half of the world's population will be overweight or obese by 2035, and approximately one-quarter of people (nearly 2 billion) will have obesity [5]. Such projections portend a steep rise in obesity-related diseases and deaths in the coming decades, underscoring the urgent need for preventive action.

The causes of this global rise in obesity are multifactorial and complex. Fundamentally, obesity develops from a chronic imbalance between energy intake and energy expenditure [[42], [43], [44], [45], [46], [47], [48], [49], [50]]. As diets worldwide have shifted toward high-calorie, processed foods and beverages, and as physical activity levels have declined due to increasingly sedentary occupations and lifestyles, many populations have experienced a sustained caloric surplus conducive to weight gain. The modern environment in both urban and rural areas often promotes overeating and inactivity – an “obesogenic” environment characterized by the easy availability of inexpensive, energy-dense foods and reduced opportunities for physical exercise [51,52]. These environmental and behavioral changes act upon underlying individual susceptibilities. Research indicates that genetic, endocrine, and metabolic factors modulate one's propensity to gain weight [53,54]. For example, genetic predisposition plays a significant role in obesity risk, influencing mechanisms of appetite regulation and fat storage [55]. However, genetic factors usually require enabling environmental conditions (such as plentiful high-fat/high-sugar diets) to fully manifest. Thus, the surge in obesity reflects an interaction between biology and environment–the modern lifestyle provides the trigger for latent genetic tendencies toward weight gain.

In addition to biological and lifestyle factors, socioeconomic and psychosocial determinants contribute substantially to the obesity epidemic. There is a well-documented inverse relationship between socio-economic status and obesity in many societies: communities with lower income or education levels often have higher obesity rates [56]. Constrained resources can limit access to healthy foods (e.g. fresh produce) and safe spaces for exercise, while cheap ultra-processed foods and sugary drinks become staples in low-income households. Moreover, chronic stress and adverse social conditions can encourage unhealthy eating behaviors [57,58]. Food is frequently used as a coping mechanism for stress or emotional distress, leading to “emotional eating” of calorie-dense comfort foods rather than eating driven by hunger. Studies have shown that psychological distress, especially when coupled with social disadvantage, can induce a vicious cycle of overeating, stress, and further weight gain [59,60]. Consequently, those facing economic hardship or psychosocial stressors may be at elevated risk of obesity. It is also recognized that certain medical conditions (such as hypothyroidism, Cushing's syndrome and polycystic ovary syndrome) and some medications can cause weight gain in susceptible individuals. While such cases account for a minority of obesity at the population level, they further illustrate the diverse array of factors that can lead to excess adiposity. Overall, obesity is best understood as a multifactorial disease – *not* simply a result of individual willpower or behavior, but rather the outcome of complex interactions among environmental, genetic, metabolic, and social influences.

The health implications of obesity are wide-ranging and profound. Excess body weight is a major risk factor for numerous chronic diseases and is associated with significantly increased morbidity and mortality. Most notably, obesity greatly elevates the risk of cardiovascular diseases such as coronary heart disease and stroke [[61], [62], [63]], which are the leading causes of death globally. Individuals with obesity are far more likely to develop type 2 diabetes, hypertension, and dyslipidemia, conditions that together contribute to the metabolic syndrome and drive cardiovascular complications [62]. In addition, obesity can lead to respiratory problems (for example, obstructive sleep apnea and obesity hypoventilation syndrome) [[64], [65], [66]] and exacerbate musculoskeletal disorders like osteoarthritis by placing excessive stress on weight-bearing joints [[67], [68], [69], [70], [71]]. Perhaps most alarmingly, obesity is strongly linked to increased cancer risk: it has been implicated in the development of common malignancies such as post-menopausal breast, colorectal, liver, kidney, and endometrial cancers [[72], [73], [74]]. The mechanisms underlying obesity-related cancers are active areas of research, but likely involve chronic inflammation, insulin resistance, and hormonal alterations caused by excess adipose tissue [75,76]. The cumulative effect of these comorbidities is that obesity substantially shortens lifespan. Epidemiological estimates attribute roughly 5 million deaths worldwide in 2019 to high body-mass index, reflecting the enormous mortality burden of obesity-related conditions [77]. In sum, obesity harms virtually every organ system and is now understood to be a leading cause of preventable illness and death in both developed and developing countries.

Beyond its toll on individual health, obesity imposes significant societal and economic costs. Healthcare systems are straining under the burden of treating obesity-linked diseases—ranging from diabetes management to cardiac surgeries and cancer therapies—diverting substantial resources to obesity-related care. The economic impact of obesity is evidenced by rising healthcare expenditures and losses in productivity due to illness and disability. Recent analyses estimate that by 2035, the global economic cost of overweight and obesity will reach approximately $4.32 trillion annually if current trends continue [78]. This staggering figure is equivalent to almost 3 % of worldwide GDP, comparable to the economic impact of the COVID-19 pandemic in 2020 [79]. Already, many countries spend sizable proportions of their health budgets on obesity and its complications, and employers face productivity losses from obesity-related absenteeism. The long-term macroeconomic drag of an unhealthy population—through increased healthcare spending and reduced workforce participation—can impede development gains, particularly in nations where obesity is rising rapidly. Thus, the epidemic of obesity is not only a medical issue but also a socioeconomic crisis, threatening to overwhelm health systems and hinder economic progress if left unaddressed.

The societal impact of obesity extends further into the psychosocial realm, affecting quality of life and social well-being. Individuals living with obesity often face weight-related stigma and discrimination, which can have severe psychological consequences [[80], [81], [82], [83]]. Negative societal attitudes portray obesity as a personal failing, leading to prejudice in workplaces, schools, and healthcare settings. Experiencing such stigma has been linked to low self-esteem, depression, and social isolation among people with obesity [84,85]. Paradoxically, weight stigma can itself reinforce the obesity problem: those who encounter weight-based discrimination or shame may avoid physical activity in public due to fear of judgment, or they may resort to coping through binge eating, thus perpetuating a harmful cycle. Research confirms that obesity stigma contributes to worsened mental health and even deters individuals from seeking medical care, thereby undermining obesity treatment and prevention efforts [85]. The psychosocial burden of obesity is especially pronounced in children and adolescents, who may suffer bullying and develop body image issues that track into adulthood. In these ways, obesity's impact on society goes beyond clinical health outcomes, also encompassing diminished quality of life, social inequities, and challenges to mental health.

In light of the overwhelming evidence of obesity's consequences, public health authorities worldwide have declared the need for urgent, multi-faceted action. Leading health organizations now recognize obesity as a chronic disease and a top public health priority. The World Health Organization (WHO) and other bodies have called for comprehensive strategies to prevent and manage obesity, including promoting healthier diets and physical activity from early childhood onward [86]. Policies targeting the environmental drivers of obesity are crucial – for example, regulating the marketing of unhealthy foods, improving urban design to encourage active lifestyles, and ensuring affordable access to nutritious foods. Indeed, at the 2022 World 10.13039/100018696Health Assembly, member states endorsed an acceleration plan for obesity prevention, aiming to support countries in implementing effective interventions by 2030 [86]. This high-level commitment reflects a consensus that without decisive action, the human and economic costs of obesity will continue to mount. As the foregoing discussion illustrates, obesity's global prevalence, its multifactorial causes, the myriad health complications it engenders, and its broad societal impact together make it one of the defining public health challenges of our time. A deeper understanding of these aspects of the obesity crisis is essential for devising informed policies and concerted efforts to curb this epidemic and improve population health worldwide.

### 1.1 Definitions of obesity

Obesity is a chronic and multifactorial disease characterized by excessive body fat accumulation, which significantly impairs health. The concept of obesity has also evolved with the knowledge that it is not just a response to excessive caloric intake but, rather, a problem involving interactions between genetic, environmental, behavioral, and metabolic factors. The most commonly used definition of obesity is based on the Body Mass Index, which, is measured by weight in kilograms divided by the square of height in metres. According to WHO, obesity is defined by a BMI at or above 30 [87]. However, the very well-known limitation of BMI is that it does not differentiate between fat mass and lean mass and thus cannot correctly indicate body fat distribution or the health risks related to obesity. As Mehrzad (2020) has indicated, the increasing criticisms against using BMI as a sole measure for obesity are because it overtly simplifies the condition, which is heterogeneous in presentation and associated health risks [88].

Recent literature has emphasized the fact that obesity is a disease process characterized by excessive adiposity and one that is associated with serious health hazards. In this regard, the American Medical Association in 2013 recognized obesity as a disease, affirming the position of the medical community that obesity is not a personal lifestyle choice but, instead, a medical condition that warrants treatment itself [89]. The ABCD framework now redefined obesity as a chronic disease process where the fundamental pathology consists of excess body fat with associated risks [90].

### 1.2 Global trends and projections of obesity

#### 1.2.1 Adult obesity

Adult obesity has reached pandemic proportions and is now a major public health concern worldwide. The World Health Organization (WHO) defines obesity as having a body mass index (BMI) of 30 kg/m^2^ or higher [87]. In 2022, an estimated 13 % of the global adult population—approximately 890 million individuals—were classified as obese. This number is projected to exceed 1 billion by 2030 [91,92]. If current trends persist, the number of obese adults is expected to grow from 810 million in 2020 to 1.53 billion by 2035 and reach 3.80 billion by 2050 [6,54,92].

The global burden of obesity is not evenly distributed and displays significant regional variations. High-income countries generally exhibit high but stable prevalence rates. For instance, in the United States, nearly 47 % of adults are obese [93]. Conversely, low- and middle-income countries (LMICs) are witnessing a rapid increase in obesity rates. In Africa, projections for 2030 indicate that 20.41 % of women and 7.76 % of men will be obese [92]. In the Eastern Mediterranean region, countries such as Kuwait, Qatar, and Saudi Arabia already report obesity rates exceeding 35 %. By 2030, it is estimated that 33.15 % of women and 21.69 % of men in this region will be obese [92]. Globally, obesity prevalence is expected to rise from 14 % to 20 % among women and from 9 % to 15 % among men by 2030 [93].

Recent data published by *The Lancet* further highlights the continued global increase in overweight and obesity from 1990 to 2021 [6]. As of 2021, an estimated 1 billion adult men and 1.11 billion adult women were affected [6]. China reported the highest number of adults with overweight and obesity (402 million), followed by India (180 million) and the United States (172 million) [6]. The most elevated age-standardised prevalence rates were found in regions such as Oceania, North Africa, and the Middle East, where over 80 % of adults in some countries were affected [6]. Compared to 1990, the global prevalence of obesity increased by 155.1 % in men and 104.9 % in women, with the most pronounced rises seen in North Africa and the Middle East—regions where male obesity rates more than tripled and female rates more than doubled [6].

Projections suggest that if these trends continue, over half of the global adult population—approximately 3.80 billion people—will be living with overweight or obesity by 2050 [6]. While China, India, and the United States are projected to remain major contributors to this burden, sub-Saharan Africa is expected to see the most dramatic growth, with a projected increase of 254.8 % in the number of adults affected. Nigeria, in particular, is forecasted to become the fourth-largest contributor by 2050, with 141 million obese adults [6].

Obesity prevalence also exhibits marked disparities by age and sex. By 2030, it is anticipated that 40 % of women and 34 % of men in the Americas will be obese, with obesity rates among middle-aged adults ranging between 25 % and 30 % [54]. In many LMICs, women bear a disproportionate burden of obesity due to a combination of cultural norms and socioeconomic factors [91]. These growing rates of obesity are expected to place increasing pressure on health systems, particularly in LMICs, where resources for prevention and management are limited. [92]. Iraq serves as a clear example of this dual burden. Currently, obesity affects 45 % of Iraqi adults and 52 % of women. Projections estimate that by 2030, approximately 47 % of Iraqi adults will be obese, adding substantial strain to the country's healthcare system. Iraq also faces the additional challenge of managing both undernutrition and rising obesity, which together contribute to elevated rates of diabetes and cardiovascular diseases (CVDs) [92].

#### 1.2.2 Childhood obesity

Childhood obesity has emerged as a significant global health concern, paralleling the rise in adult obesity and contributing to a lifetime trajectory of poor health. Recent estimates suggest that in 2022, approximately 159 million children and adolescents worldwide were living with obesity, with forecasts predicting that this number will increase to 254 million by 2035 and 390 million by 2050 if current trends persist [5,94]. This alarming rise has been particularly steep in low- and middle-income countries, where nutrition transitions, urbanization, and increased consumption of ultra-processed foods have driven rapid shifts in childhood weight status [5,[95], [96], [97]].

The consequences of childhood obesity are both immediate and long-term. In the short term, children with obesity are at greater risk for early-onset metabolic disorders, such as insulin resistance, type 2 diabetes, dyslipidemia, and hypertension, which were previously considered adult conditions [98]. They may also experience orthopedic problems, nonalcoholic fatty liver disease, and psychological distress, including low self-esteem and social stigma [99]. A recent studies have shown that children with obesity are significantly more likely to remain obese into adulthood and to develop non-communicable diseases (NCDs) at younger ages [[100], [101], [102]].

Prevention and management strategies for childhood obesity must be multifaceted and age-appropriate. School-based interventions have demonstrated effectiveness in promoting healthy behaviors, particularly when they include nutritional education, structured physical activity, and healthy food provision in cafeterias [103]. For example, multi-component programs such as the WHO's Health Promoting Schools framework emphasize integrating health into the school curriculum, policies, and environment [104]. Family-based interventions that engage caregivers in meal planning, physical activity, and behavioral support are also crucial, given the strong influence of home environments on children's dietary habits [[105], [106], [107], [108], [109], [110], [111]]. Moreover, policy-level measures—such as restricting the marketing of unhealthy foods to children, implementing sugar-sweetened beverage taxes, and ensuring access to safe play areas—are essential for creating supportive environments for healthier child growth trajectories [112].

### 1.3 Types of obesity

It is essential to note that obesity is not a single condition but rather occurs in several forms. Being able to identify these different types has the potential to enable the development of more focused and effective forms of treatment. In this part, I described the different types of obesity in adults, which have been classified according to factors such as distribution of fat, metabolic syndrome, genetics, and content of muscle-fat.

#### 1.3.1 Generalized (overall) obesity

Generalized obesity is the generalized increase in body fat, which is evenly distributed in the body, for which the BMI usually diagnoses it. A BMI of 30 kg/m^2^ or more will usually diagnose obesity [113]. This kind of obesity predisposes an individual to a number of comorbidities, such as type 2 diabetes, heart disease, and cancer. While being a general method of diagnosis for generalized obesity, BMI fails to capture the risk that every form of fat distribution creates; hence, more specific forms of obesity types have been identified.

#### 1.3.2 Central (visceral) obesity

Central obesity can also be referred to as visceral or abdominal obesity. It involves an accumulation of fat around the abdominal area in each internal organ. It has strong associations with metabolic syndrome, cardiovascular diseases, and insulin resistance. This condition usually occurs when waist circumference is above 102 cm for men and 88 cm for women [114]. Visceral fat is one form of fat that is metabolically active, secreting inflammatory cytokines, enhancing insulin resistance, and thereby predisposing individuals to chronic diseases, especially cardiovascular conditions [115].

#### 1.3.3 Subcutaneous obesity

Subcutaneous obesity refers to the deposition of extra fat just under the skin, usually in the hips, thighs, and buttocks. Though it contains extra fat, due to lower metabolic activity, subcutaneous fat is considered less dangerous compared to visceral fat. Moreover, individuals with subcutaneous obesity can also have a high risk for health complications if the total percentage of body fat is too high. This type of obesity is more common in females and less strongly associated with metabolic diseases than central obesity [114].

#### 1.3.4 Metabolically Healthy Obesity (MHO)

Metabolically Healthy Obesity (MHO) describes individuals who are classified as obese according to their BMI but do not exhibit common obesity-related metabolic disorders, such as insulin resistance, hypertension, or dyslipidemia. Studies show that above 30 % of obese individuals may fit into this category [116]. Although these individuals may initially appear to be at lower risk for chronic diseases, recent research suggests that MHO is not entirely benign. Over time, many individuals with MHO may develop metabolic issues, particularly if preventive measures such as diet and exercise are not implemented [117].

#### 1.3.5 Metabolically obese, normal weight (MONW)

MONW is a medical condition wherein a person with normal weight, with a BMI of 18.5–24.9 kg/m^2^, may have metabolic risk factors similar to those possessed by obese individuals, such as high blood sugar, a high level of cholesterol, and hypertension. Individuals who have the usual body weight may also have too much visceral fat that puts them at an increased risk for cardiovascular diseases and type 2 diabetes [118]. Individuals with MONW are usually overlooked in the typical clinical setting because of their normal appearance of BMI. This calls for the reason behind the assessment of metabolic markers even in those people who do not meet the traditional definitions of obesity [119].

#### 1.3.6 Genetic obesity

A great contribution to obesity can be made by genetic factors. Probably the most well-known genetic factor, the FTO gene stands for Fat mass and obesity-associated gene. It has been associated with higher body mass index and a greater risk of developing obesity. Individuals possessing certain variants of the FTO gene may be at heightened risk of gaining weight through childhood and adult years. Out-of-control genetic obesity is normally difficult to manage since it usually relates to innate factors that place a living organism at risk of excessive weight gain. Nonetheless, environmental factors such as diet, exercise, and lifestyle determine the magnitude at which genetic tendencies develop into obesity [120,121].

#### 1.3.7 Sarcopenic obesity

Sarcopenic obesity refers both to age-associated loss of muscle mass and strength combined with obesity. This form of obesity is increasingly present in older adults and presents different health challenges, given the combination of the physical limitations associated with sarcopenia and metabolic risks from excess body fat. Sarcopenic obesity increases the risk to frailty and falls, further leading to decreased mobility and metabolic complications such as insulin resistance and cardiovascular diseases. Special attention must be given to the treatment of sarcopenic obesity because traditional weight loss, in general, focuses only on fat reduction and may further promote muscle loss. Treatments should, therefore, be directed at fat reduction together with the preservation or increase of muscle mass through resistance training and appropriate nutrition [122,123].

#### 1.3.8 Hormonal obesity

Different forms of hormonal imbalances and endocrine disorders can lead to obesity. For example, hypothyroidism, Cushing's syndrome, and polycystic ovary syndrome may all lead to metabolic dysfunctions that in turn commonly result in weight gain. Typically, obesity resulting from endocrine conditions is characterized by specific patterns of fat distribution, such as central obesity, and specific treatments are often required, including hormone therapy or medication to manage the underlying condition [124,125].

### 1.4 Obesity calculations

#### 1.4.1 Body mass index (BMI)

**Fig. 1:**
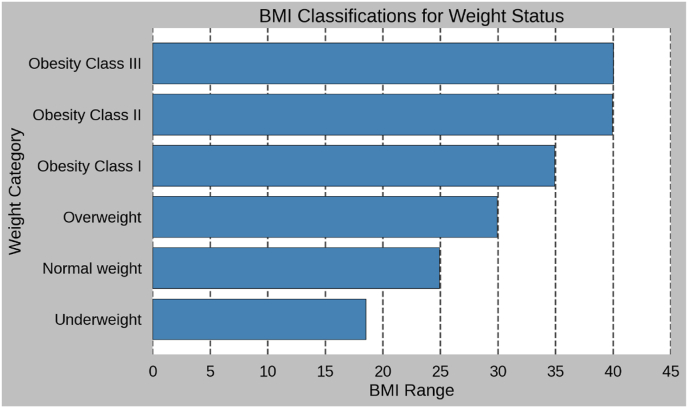
Body Mass Index (BMI) classifications for weight status [87].

The most widely used tool to assess obesity is the BMI. Very simple calculation based on weight and height, defined as weight in kilograms of a person divided by height in meters squared-BMI = kg/m^2^v (Fig. 1).

Where BMI does serve well as a useful screening tool, it is highly limited in capturing the complexity of obesity. For example, individuals with the same BMI can have completely different body fat contents and health risks depending on age, gender, ethnicity, and fat distribution. Because of this, researchers are calling for more sophisticated classification systems that take into consideration distribution of fat-visceral versus subcutaneous fat-metabolic health, and comorbid conditions [126,127].

#### 1.4.2 Body fat percentage (BFP)

BFP is a more direct measurement of body composition than is BMI. It is an estimation of what percentage of a person's body mass consists of fat. A number of techniques for measuring body fat are available, including bioelectrical impedance analysis (BIA), dual-energy X-ray absorptiometry (DXA), and skinfold measurement. All these techniques are considered more accurate measurements of obesity than is BMI, since they actually measure fleshly mass against bone and muscle. Levels of body fat exceeding 25 % in men and 33 % in women are normally considered to be indicative of obesity in adults. This measurement of body fat is very accurate, but because it requires specialized equipment and trained personnel, its use is less practical than the use of BMI in large-scale epidemiological studies [128].

#### 1.4.3 Waist circumference (WC)

WC is the measurement of central adiposity, which is very closely related to visceral fat-the main type of fat stored around internal organs, where metabolic risk increases. Thus, even with a normal BMI, studies have been able to demonstrate that individuals with excess visceral fat are more susceptible to conditions such as type 2 diabetes and cardiovascular diseases [129].

For men, a waist circumference greater than 102 cm (40 inches) and for women, greater than 88 cm (35 inches), indicates a higher risk for obesity-related health problems [130]. Measuring waist circumference is a simple, non-invasive, and inexpensive procedure; thus, it is commonly used in both clinical and epidemiological environments. However, WC does not take into consideration general body fat distribution and may be subject to factors such as bloating or posture at the time of measurement.

#### 1.4.4 Waist-to-hip ratio (WHR)

The WHR is another technique that takes into account the distribution of fat. That is, the ratio of the circumference of the waist is compared to that of the hips [131]. The measurement can indicate central obesity and is, thus, considered one of the major risk factors for cardiovascular diseases. Normally, a WHR above 0.90 for men and 0.85 for women is regarded as a sign of abdominal obesity [132]. Similar to waist circumference, WHR is an easy and inexpensive approach; however, it also has some disadvantages. For example, it does not provide the absolute measure of body fat but rather illuminates the distribution of fat that may not account for generalized obesity.

#### 1.4.5 Waist-to-hight ratio (WHtR)

Waist-to-Height Ratio is a simple yet highly effective tool in the assessment of central obesity and cardiometabolic risk. In fact, studies have indicated that WHtR can be used as a better predictor of cardiometabolic risk factors than the other anthropometric measures of BMI and waist circumference alone, especially because the latter takes into consideration fat distribution around the abdomen-an important factor in metabolic health. Hence, a WHtR of over 0.5 is generally considered as reflective of higher risk. Indeed, evidence of these facts shows that WHtR can be applied across different age, sex, and ethnic groups, and is flexible as well as a reliable measure for clinical assessments [133,134].

#### 1.4.6 Relative fat mass (RFM)

RFM is the more modern method used to approximate the quantity of body fat based on height and waist size [135]. This was developed as an alternative approach to BMI, addressing the limitations that this latter has in differentiating between fat and muscles. RFM also provides a more direct estimation of body fat and has been found to be more precise in predicting risks due to obesity, especially when compared to the use of BMI [136]. RFM remains a relatively new measure, and further studies will be needed for its full validation in diverse populations.

#### 1.4.7 Bioelectrical impedance analysis (BIA)

Bioelectrical impedance analysis is a non-invasive measurement of body composition in which a small, harmless electrical current is introduced into the body tissues to estimate body composition by measuring the resistance of body tissues to an electrical current. The principle behind BIA is that lean tissues are more conductive of electricity than fatty tissues due to the greater water content. BIA is quick and non-invasive and, therefore, convenient for field and clinical use [137].

### 1.5 Factors affecting of obesity development

**Fig. 2:**
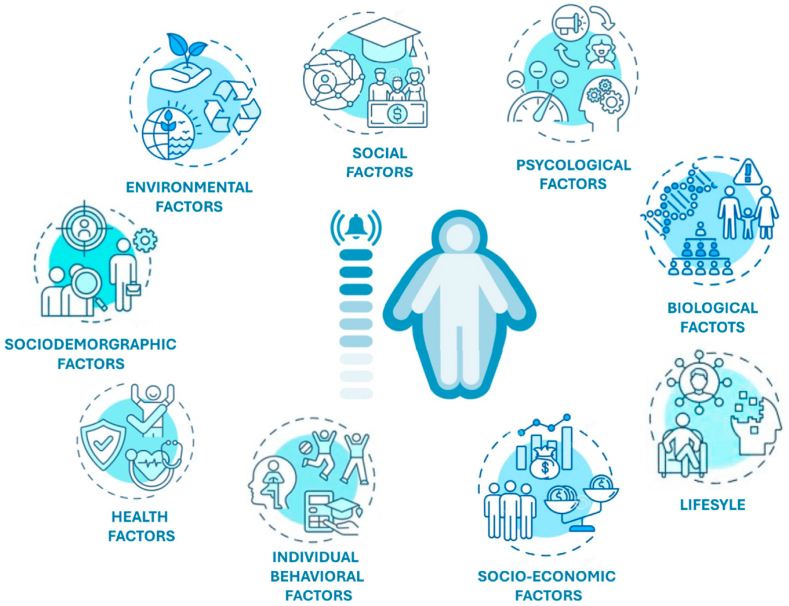
The principal factors leading to obesity. source [138] licensed under CC BY 4.0.

The various factors that have been contributing to the obesity epidemic represented in Fig. 2.

#### 1.5.1 Biological factors of obesity

Biological factors are one of the causes of obesity, which includes genetics [139]. It is estimated that up to 70 % of variability in body weight is due to genetic factors [140]. This includes genetic variants that exist, such as the FTO gene, which can provide a predisposition toward an increase in appetite with less satiety, hence increased consumption of calories [141]. Even this genetically set functioning is actually tuned epigenetically, for instance, through nutrition. These changes could contribute to a difference in metabolism and fat deposition, hence increasing the risk for obesity [142].

Other critical factors in obesity include metabolic dysfunctions. People with lower basal metabolic rates have a tendency to store more fat, thereby becoming vulnerable to weight gain. Insulin resistance, common among obese individuals, promotes fat storage through excessive deposition, especially in the abdominal area. Such a state of metabolic disequilibrium highly influences many diseases that come hand in hand with obesity, such as type 2 diabetes and cardiovascular disorders [143].

At the same time, not less crucial for obesity is hormonal regulation. Usually, leptin suppresses appetite; via leptin resistance, it becomes less effective in obese individuals, which causes overeating [144]. The level of ghrelin is normally higher in obesity-a hormone that acts to stimulate appetite-causing a greater feeling of appetite [145]. This generally makes weight regulation quite hard for those who have it. Besides this, gut microbiota has become one of the major factors contributing to obesity [146]. Generally, the gut microbiome in obese persons differs from that in people of normal weight due to its higher ratio of Firmicutes to Bacteroidetes, facilitating better energy extraction from food [147]. This disturbance in the microbial community encourages fat deposition and low-grade chronic inflammation, which may result in further disturbance of metabolic processes [148].

#### 1.5.2 Behavioral factors of obesity

Behavioral factors play a big role in problems with obesity mainly through lack of physical activity, unhealthy eating patterns, and emotional eating [147,149]. The modern societies are conducting the sedentary way of life and greatly decreased daily energy expenditure. This is further enhanced by an environment that supports physical inactivity such as long hours of sitting at workplaces, lack of easy access to recreational places, and increased utilization of vehicles for travelling [[150], [151], [152]]. It has been highlighted that the general factor of physical inactivity is highly associated with gaining weight and developing obesity because it limits energy utilization and increases fat deposition [152].

**Fig. 3:**
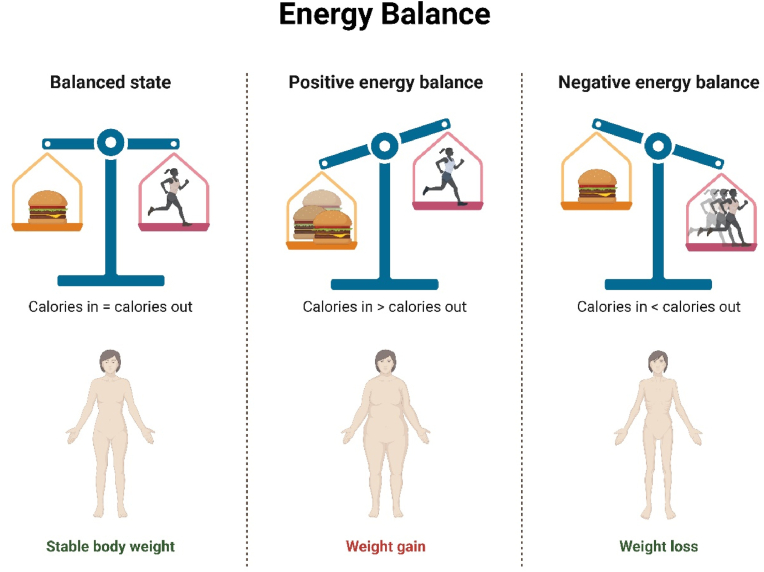
Energy balance and its impact on weight regulation.

The second major factor contributing to obesity is unhealthy dietary habits, especially the excessive consumption of foods that are high in calories and low in nutrients. High-fat, high-sugar foods are pervasive and heavily marketed; this stimulates overeating, generally leading individuals to take in more calories than they actually need [153,154]. This pattern is compounded by large serving sizes and the tendency to equate snacking with highly processed foods. Studies have recorded that persons who consume a lot of food from fast foods, sweetened beverages, and snacks that contain a high content of processed ingredients run a very high risk of developing obesity [138]. Fig. 3 explains the concept of energy balance and its effect on body weight, comparing three states: balanced, positive, and negative energy balance. In a balanced state, calorie intake equals calorie expenditure, leading to stable body weight. A positive energy balance occurs when calories consumed exceed calories burned, resulting in weight gain, while a negative energy balance, where calories burned exceed intake, leads to weight loss (Fig. 3).

Other critical behavioral factors involve emotional eating, where one eats in response to negative emotions, such as stress, anxiety, or even sadness. This leads to overeating and overindulgence in comfort foods, which are usually highly loaded with sugar and fat. Poorer emotional regulation and resorting to poor coping mechanisms for dealing with stress have also been associated with this behavior [155]. Research has provided strong evidence for the positive association of emotional eating with weight gain, particularly in individuals who are suffering from depression or anxiety [60]. In fact, emotional eating is one of the most prominent reasons people regain weight after dieting when they return to their former, unhealthy eating patterns.

Chronic stress also plays a major role in influencing the behavioral patterns that lead to obesity [156]. Stress activates the HPA axis, which leads to the overproduction of cortisol, a hormone related to encouraging fat deposition, particularly around the central part of the body [157]. Stress is also linked with an increased desire for high-calorie, palatable foods because such food is usually used as a coping mechanism to help lower stress levels [157]. This has created a vicious circle where stress leads to overeating, further encouraging weight gain and making it hard for individuals to maintain a healthy weight.

#### 1.5.3 Environmental factors of obesity in adults

The environmental factors are major contributors to increasing rates of obesity in the world today. Among the most critical environmental influences on obesity, those related to build environment stand at the top [158,159]. Urbanization came along with changing city planning and, consequently, led to lower levels of physical activity due to more people living in areas without easy access to parks and recreational facilities or places that can be used safely for walking [160]. For example, it has been documented that urban sprawl with limited green space and fewer transportation options can cut down daily exercise time significantly and thus cause greater weight gain [161]. Areas where walkability is greater or where recreational facilities are more accessible have higher records of lesser obesity rates; thus, indicating how urban planning can result in healthy living [162].

**Fig. 4:**
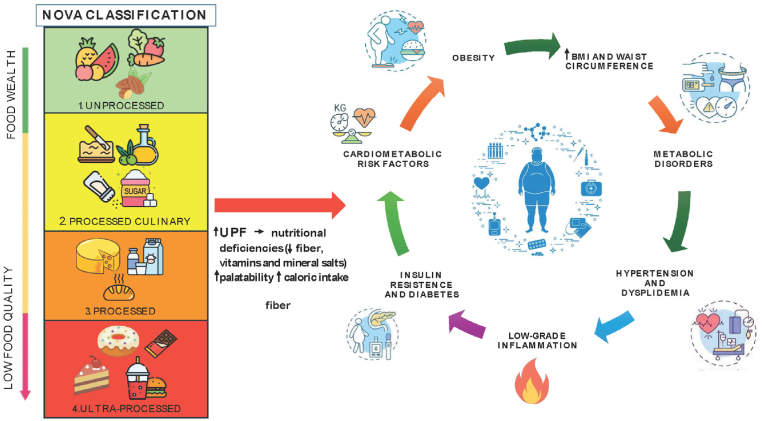
The relationship between Ultra-Processed Food (UPF) foods and obesity. Source [138] licensed under CC BY 4.0.

The nutritional environment is another formidable environmental factor. Over the past decades, foods that are nutritionally unhealthy and calorie-dense became much more accessible, available, and affordable. Often, heavily marketed and cheaply sold foods contain high levels of fats and sugars, whereas healthier foods are more expensive, making these products more accessible to lower-income parts of the population. It's a shift that has helped create an atmosphere in which overeating is encouraged, hence too many calories are taken in, and obesity results. Research has underlined just how much food marketing and the pervasive availability of fast food breed less-than-healthy eating and weight gain patterns, especially in neighbourhoods where healthier options are not as accessible [138] (Fig. 4).

The socioeconomic variables also play a vital role in obesity. The lower-income population is more vulnerable due to the fact that they have a low availability of healthy food stores and recreational areas [163]. Lower economic communities also tend to have much higher levels of life stress and food security that promote unhealthy eating and physical inactivity. Researchers have established that socioeconomic deprivation and population density, along with the lack of availability of fresh produce and recreational facilities, highly contribute to the prevalence of obesity in urban areas [164]. This sets up a self-perpetuating circle where there is a strong link between poverty and obesity since it becomes very difficult for the person to make healthier lifestyle choices [165].

#### 1.5.4 Psychological and social factors of obesity

Psychological and social factors highly contribute to the development and persistence of obesity. A key factor on the psychological side is emotional distress, often associated with overeating. Most people use food as their coping mechanism to overcome stress, anxiety, or depression through emotional eating. In such emotional eating, individuals eat not because of hunger but to suppress an avoidance behavior from unwanted feelings. Again, this maladaptive coping strategy almost always involves high-calorie, energy-dense foods and thus results in weight gain. Research has shown that emotional distress, when combined with socio-economic disadvantages, induces a vicious circle of emotional eating, stress, and further weight gain [166].

Another important factor of psychological burden of obesity is the weight-related stigma. In general, people with obesity may be stigmatized, that then often can develop into low self-esteem, social withdrawal, and even chronic stress [2]. This stigma can also lead to worsening psychological distress and unhealthy behaviors, such as binge eating or avoiding physical activity because of fear of judgment in public settings. Weight stigma has been associated with increased levels of cortisol and other stress responses; these, in turn, are said to promote fat storage and weight gain [167]. This becomes a cyclical relationship in that it creates a feedback loop of sorts: weight stigma, stress, and obesity.

Other social factors involve support systems and familial relationships that could also serve as a factor in obesity. Researchers have documented that individuals from socioeconomic disadvantages, or those with limited social support, are at an increased risk for psychological distress and emotional eating [168]. For example, growing up in a disharmonious family environment characterized by stress, conflict, or low social cohesion may heighten the risk of developing maladaptive eating behaviors, low self-esteem, and depression-all components of weight gain [169].

#### 1.5.5 Medical and pharmaceutical factors of obesity

One of the major contributors to obesity includes medical and pharmaceutical ones. Generally, hypothyroidism, Cushing's syndrome, and PCOS are some of the endocrine disorders shared in inducing weight gain [124]. The underproduction of thyroid hormones that occur with hypothyroidism generally lowers one's metabolic processes, eventually leading to increased weight, fatigue, and reduced fat-burning capability. Indeed, studies have documented the close link between hypothyroidism and obesity, especially in women, who are said to be more susceptible to thyroid imbalance [170]. However, many such instances may be improved upon with the treatment of hypothyroidism with the use of hormone replacement therapy, which regulates one's metabolism and, in turn, may cause weight loss [171].

The other major medical cause of obesity is Cushing's syndrome, a condition attributed to overproduction of cortisol from either endogenous overproduction or the use of corticosteroid medications over an extended period. Increased cortisol levels enhance visceral fat accumulation, especially in the abdomen, along with other signs like muscle wasting and hypertension. Among patients with Cushing's syndrome, there is often extensive central obesity; the resolution of the cause, such as a reduction in corticosteroid use or treatment of a pituitary tumor leading to overproduction of cortisol, may be associated with dramatic weight loss [172].

Another prevalent endocrine disorder is Polycystic ovary syndrome (PCOS), which is found to be associated with obesity in the majority of women afflicted by it. PCOS results in hormonal disturbance, insulin resistance, and hyperandrogenism, all of which favor weight gain, notably in the visceral area. In this regard, the pathogenesis of obesity can, in turn, accentuate the clinical features of PCOS by entering into a vicious circle of sexual hormone disturbance and weight gain. Lifestyle modification, diet, and medication like metformin that may improve insulin sensitivity could be helpful in weight loss among women with PCOS [173].

Another contributing factor to obesity may be drugs that are used for the treatment of other diseases. Most prescribed antidepressants, including the commonly used SSRIs, mood stabilizers such as lithium, and antipsychotics, are well associated with weight gain [174]. These drugs affect appetite regulation and metabolism, often causing increased intake of calories with reduced energy expenditure. Long-term corticosteroids administration, generally used for inflammatory and autoimmune disorders, results in increased fat deposition, especially around the face, the abdomen, and the upper back [175]. Reduction of dosage or withdrawal of drugs often are management options with low-weight-gain potential drugs.

#### 1.5.6 Cultural and regional variations in obesity drivers

Obesity prevalence and its underlying determinants vary significantly across regions, reflecting the interplay between cultural, economic, and environmental factors. Cultural beliefs, traditional diets, and social norms strongly influence obesity risk and shape the effectiveness of intervention strategies. In the Middle East, for example, rapid economic development, urbanization, and a shift from traditional diets to high-calorie, processed foods have driven a marked increase in obesity rates—particularly among women, who often face barriers to physical activity due to gender norms [176]. A recent studies indicated that obesity prevalence among adults exceeds or nearly 40 % in countries such as Kuwait, Qatar, and Saudi Arabia, reflecting both dietary changes and sociocultural limitations on mobility [[177], [178], [179]].

In contrast, Southeast Asian countries such as Vietnam and Indonesia have traditionally maintained plant-rich diets and high physical activity levels through agrarian lifestyles. However, economic transitions and urban sprawl have introduced Western dietary influences and sedentary behaviors, contributing to rising obesity trends, especially in urban youth populations [180]. Cultural perceptions of body size in some Southeast Asian societies may also delay recognition of obesity as a health risk, as larger body types are sometimes associated with wealth or health.

Sub-Saharan Africa presents a unique dual burden: undernutrition coexists with rising obesity in urban settings. In some African cultures, larger body size is still associated with prosperity, fertility, or beauty, which may inadvertently promote overeating or discourage weight-loss behaviors [181]. However, these perceptions are changing in younger generations, especially as awareness of non-communicable disease risks grows. Urbanization in African capitals has led to greater availability of cheap, energy-dense foods and limited access to recreational spaces, promoting obesogenic environments [[182], [183], [184], [185]].

In Western nations, obesity is more often linked to socioeconomic disparities. Low-income populations face greater exposure to ultra-processed foods due to food deserts and often lack time or resources for physical activity. Social determinants, including education and healthcare access, have a strong impact on obesity-related outcomes [186].

Therefore, effective obesity interventions must be tailored to regional and cultural contexts. For example, in the Middle East, culturally sensitive women-only fitness programs have proven successful, while in Southeast Asia, policies that promote active transportation and protect traditional diets show promise. Recognizing and integrating cultural practices into intervention design enhances community acceptance and sustainability. Global strategies should thus be flexible and region-specific, balancing universal principles with local adaptations.

#### 1.5.7 Molecular and cellular mechanisms underlying obesity

At the molecular level, obesity is underpinned by complex dysfunctions in adipocyte biology, metabolic signaling, immune activation, and gene regulation. Excess nutrient intake and sedentary behavior drive adipocyte hypertrophy and hyperplasia, particularly in visceral fat depots. This triggers a cascade of cellular stress responses that ultimately lead to chronic low-grade inflammation, hormonal imbalance, and systemic metabolic disruption.

In obesity, expanding adipose tissue becomes hypoxic and dysfunctional. Hypertrophic adipocytes secrete excess pro-inflammatory adipokines such as tumor necrosis factor-alpha (TNF-α) and interleukin-6 (IL-6), while beneficial adipokines like adiponectin are suppressed [187]. Adiponectin enhances insulin sensitivity and fatty acid oxidation, but its levels are inversely correlated with fat mass. Conversely, leptin—normally responsible for satiety signaling—rises with fat accumulation but often becomes ineffective due to central leptin resistance, contributing to persistent hunger and overeating [188].

As adipocytes enlarge, immune cells—particularly M1 macrophages—infiltrate the tissue, releasing additional cytokines and sustaining a chronic inflammatory state [189]. This inflammation exacerbates insulin resistance and impairs mitochondrial function. Mitochondrial stress in obese tissues reduces oxidative phosphorylation and increases reactive oxygen species (ROS), which further disrupt insulin signaling and lipid metabolism [190]. Mitochondrial biogenesis is also reduced, especially in skeletal muscle, diminishing the ability to utilize fatty acids efficiently [191].

Emerging evidence shows that obesity is associated with epigenetic alterations, including DNA methylation, histone modification, and microRNA regulation, which influence gene expression patterns involved in metabolism, adipogenesis, and inflammation [192]. Early-life exposures, such as maternal diet and intrauterine growth, can epigenetically program susceptibility to obesity later in life. These modifications are reversible and may present novel therapeutic targets [193].

The gut microbiome plays a critical role in nutrient absorption, energy harvest, and immune modulation. Obese individuals often exhibit a higher Firmicutes-to-Bacteroidetes ratio, which promotes increased caloric extraction from food and facilitates lipid storage [194]. Microbial metabolites like short-chain fatty acids (SCFAs) also affect host metabolism, appetite regulation, and systemic inflammation. Dysbiosis may impair gut barrier integrity, enabling translocation of lipopolysaccharides that activate inflammatory pathways and worsen metabolic endotoxemia [195]. Understanding these interconnected mechanisms provides deeper insight into the pathophysiology of obesity and may inform the development of targeted therapies, including anti-inflammatory agents, mitochondrial modulators, epigenetic drugs, and microbiota-directed interventions.

### 1.6 Health consequences of obesity

**Fig. 5:**
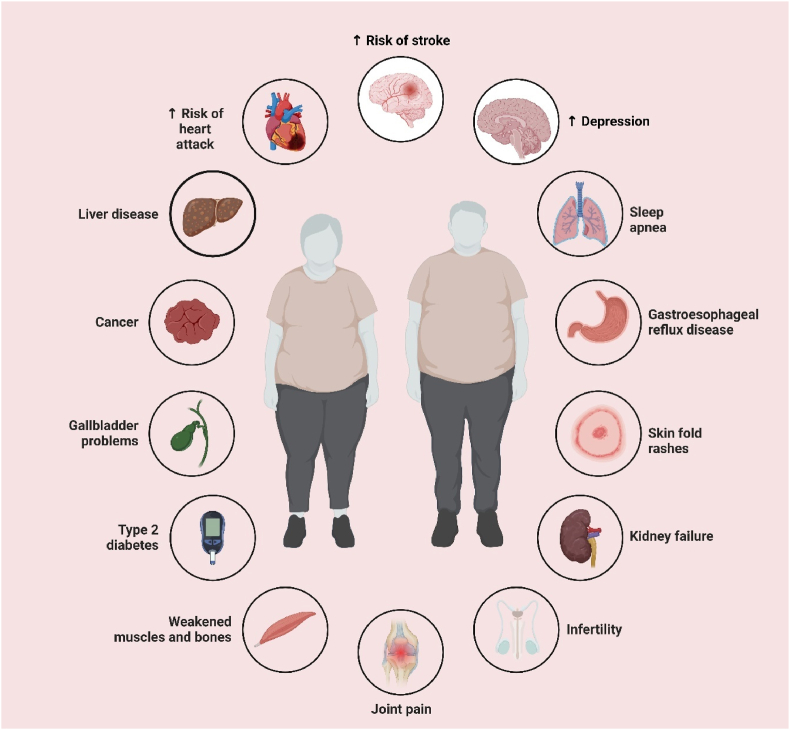
Highlights the extensive impact of obesity on various organs.

Obesity comorbidities may include cardiovascular risks such as heart attack and stroke, metabolic disorders such as Type 2 diabetes and liver disease, cancer, and kidney failure. Obesity further contributes to osteoarthritis and pain in the joints, infertility, sleep apnea, depression, and skin fold rashes. Fig. 5 shows a wide range of such health issues (Fig. 5).

#### 1.6.1 Cardiovascular consequences of obesity

One of the most accessible and earliest cardiovascular effects of obesity is hypertension [61]. The excess weight increases blood volume and cardiac output, conditions that place greater stress on the heart [196]. Moreover, obesity activates the renin-angiotensin-aldosterone system (RAAS) and the sympathetic nervous system, which elevate blood pressure even further. Chronic hypertension among obese subjects commonly leads to left ventricular hypertrophy, thereby increasing the risk of heart failure in them [197].

There is a very close relation between CAD and obesity due to the unyielding process of systemic inflammation and atherosclerotic plaques building up in the arteries. What is more important, abdominal obesity normally acts as one of the major driving forces for developing CAD, managing to enhance visceral fat deposition, which is very harmful and increases cardiovascular risks [62].

The other major result of obesity is heart failure. The excess weight puts the heart under increased pressure, which contributes to both systolic and diastolic dysfunction. Obesity-related heart failure is on the rise, considering the rates of obesity itself are improving along with its associated metabolic disturbances, such as insulin resistance [198].

Obese conditions serve as a source for both ischemic and hemorrhagic stroke due to the combined effects of hypertension, atherosclerosis, and the pro-inflammatory state contributed by obesity [199]. Obesity enhances clot formation and thereby increases the risk of ischemic stroke, while high blood pressure raises the risk of hemorrhagic stroke [62].

#### 1.6.2 Metabolic disorders consequences of obesity

Obesity is closely associated with type 2 diabetes because excess body fat promotes insulin resistance. When cells become resistant to insulin, the uptake of glucose into the cells becomes impaired, leading to increased blood sugar levels and a greater risk of diabetes. Weight loss has been shown to significantly improve insulin sensitivity and, therefore, decrease the risk of developing type 2 diabetes [200].

Metabolic syndrome is characterized by central obesity, hypertension, and dyslipidemia. It is very common in obese subjects and powerfully predicts cardiovascular disease. NAFLD is considered the hepatic manifestation of metabolic syndrome and is very common in this population [201].

Dyslipidemia is commonly featured in obesity; it consists of a high level of triglycerides and low HDL cholesterol. Such dysregulation further predisposes an individual to cardiovascular disease and is closely associated with obesity, especially when there is deposition of abdominal fat. Weight reduction improves the lipid profile and decreases the risk of complications arising from dyslipidemia [202].

NAFLD is a liver disease strongly associated with obesity. It encompasses a disease spectrum extending from simple fatty liver to cirrhosis. NAFLD enhances insulin resistance and fuels the development of metabolic syndrome and type 2 diabetes [203].

#### 1.6.3 Respiratory and sleep related consequences of obesity

Obese-related factors are a few of the most important risk factors for respiratory and sleep-related issues. Of these, the most important conditions include obstructive sleep apnea (OSA), obesity hypoventilation syndrome (OHS), and asthma.

Obstructive Sleep Apnea (OSA) is very common in people suffering from obesity. It results from fat accumulation around the neck and uppermost airway, obstructing the air passage during sleep [204]. This condition is manifested by partial or complete cessation of breathing. It is also commonly associated with excessive daytime sleepiness, fatigue, and a very high risk of cardiovascular diseases. Weight reduction has been found to enhance OSA symptoms and overall respiratory function [205].

Obesity Hypoventilation Syndrome (OHS) is defined by the presence of obesity in combination with daytime hypercapnia and sleep-disordered breathing. The majority of these patients do have OSA, but it is not a requirement for diagnosis. The hypoventilation that often characterizes OHS during sleep results in chronic respiratory failure if the condition is left untreated. Treatment of OHS generally focuses on the use of PAP therapy, often in conjunction with attempts at weight management, as symptoms can be considerably improved with weight loss [206].

Asthma also tends to be higher in obese individuals, and indeed studies have proved that excess weight enhances symptoms of asthma, hence leading to poor respiratory outcomes. The mechanisms involved are not yet fully comprehended, but it is believed that inflammation, mechanical changes in pulmonary function, and airway responsiveness play key roles. Moreover, weight loss has been able to reduce asthma symptoms in obese patients, reinforcing the thought that management of body weight is principal in respiratory health [207].

#### 1.6.4 Musculoskeletal consequences of obesity

Obesity is known to be a risk factor for several musculoskeletal disorders, like osteoarthritis, lower back pain, and gout. This excess weight places greater stress on the joints, tendons, and bones, hastening wear and tear processes, especially in weight-bearing joints such as the knees and hips [208].

Osteoarthritis is the most common musculoskeletal disease associated with obesity. In addition to the mechanical load on the joints, the increase in body weight promotes the breakdown of cartilage and the inflammatory pain. Recently, the low-grade inflammation characteristic of obese individuals was also thought to accelerate further the course of OA, making this condition not strictly a mechanical problem but also a metabolic one [68]. Weight loss has been shown to improve symptoms of OA, especially in the knee, where osteoarthritis related to obesity is most prevalent [209].

The other common musculoskeletal condition in the obese population is lower back pain. The excess weight modifies the biomechanics of the spine and increases the stress exerted on the intervertebral disc and muscles supporting the back [210]. Obesity also accelerates intervertebral disc degeneration, one major causative factor for chronic back pain [211].

Besides, gout is another form of inflammatory arthritis that is also very strongly associated with obesity. With a high body mass index, uric acid production increases, followed by crystallization of the substance in joints, thus triggering painful gout attacks. It has been found from study that management of obesity-which involves reducing weight and changing eating habits-can be responsible for less frequency and severity in the flare-ups of gout [212].

#### 1.6.5 Cancer related obesity

**Fig. 6:**
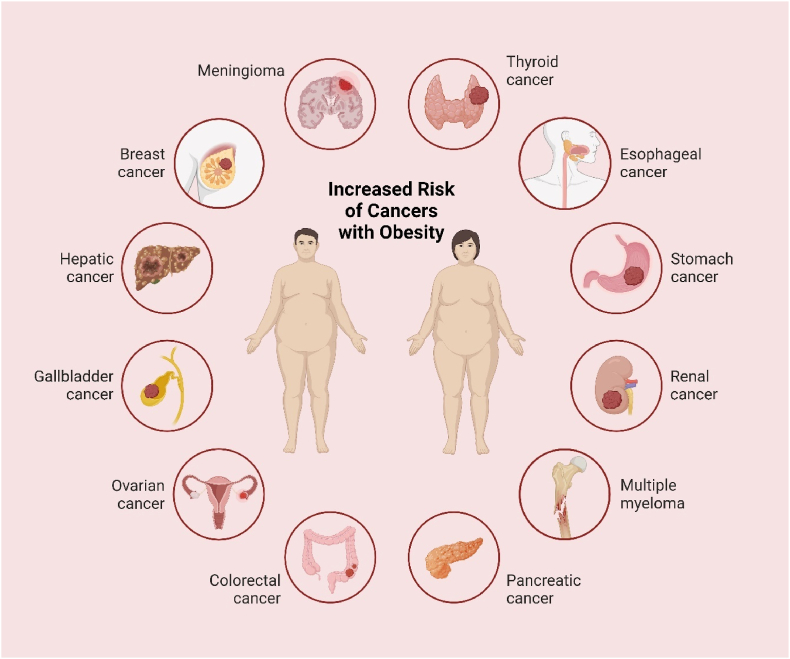
Highlights Major cancers associated with obesity.

Obesity is also known to raise the risk for a variety of cancers, hence being a serious public health concern (Fig. 6). In general, mechanisms for obesity leading to cancer are complex and multifactorial in nature, including chronic inflammation, hormonal imbalance, and insulin resistance.

In postmenopausal women, obesity increases the likelihood of a new diagnosis of breast cancer. Extra body fat exposes a person to higher levels of estrogen, which can feed the growth of hormone-receptor-positive breast cancers. Obesity has also been linked with poorer prognosis and increased mortality among women diagnosed with breast cancer [73]. It is known that obesity raises the amount of estrogen produced by adipose tissues, thereby increasing the risk of endometrial cancer [213]. In fact, obese women have three times the risk of developing endometrial cancer when compared to normal body mass index [214]. Abdominal obesity, due to normally resultant inflammation and insulin resistance, is a strong risk factor for colorectal cancer. Visceral fat may contribute to a pro-inflammatory state that favors tumor growth in the colon and rectum [215].

Obesity heightens the risk of pancreatic cancer by contributing to insulin resistance and higher circulating levels of insulin, which are then associated with the development of tumors in the pancreas. Obesity also increases the mortality rate in individuals affected with pancreatic cancer [73]. Additional body fat raises the risk for renal cancer through alterations in insulin signaling pathways and by promoting inflammation; both factors encourage tumor growth. Obesity has a strong association with renal cell carcinoma [216]. Since obese individuals have a higher tendency to develop gallstones, these are recognized as major risk factors for gallbladder cancer. Chronic inflammation and hormonal imbalance associated with obesity contribute to the gallbladder carcinogenesis [217]. More precisely, abdominal obesity enhances the risk for obesity because of the higher incidence of gastroesophageal reflux disease in individuals with excess body fat. Chronic GERD may lead to a premalignant condition of the esophagus known as Barrett's esophagus [218]. It is also known that obesity is an important factor in the development of NAFLD, which can further lead to liver cirrhosis and hepatocellular carcinoma. The combination of insulin resistance, chronic inflammation, and fat accumulation in the liver makes the obese host very prone to liver cancer [219]. Although less established, obesity is suggested to be related to an increased risk of ovarian cancer via hormonal pathways and chronic inflammation. Studies have also indicated a higher risk for obese women compared with those with normal weight [220].

### 1.7 Management and prevention strategies of obesity

#### 1.7.1 Behavioural interventions for management of obesity

Behavioral interventions play a vital role in the management of obesity through lifestyle modification for long-term weight loss and maintenance [221]. Dietary modification is one of the most important components of such behavioral interventions, which essentially involves a reduction in calorie intake with an assurance to provide a nutrient-dense diet. Dietary programs that are individually tailored, meeting needs and preferences-low-carbohydrate or Mediterranean diets, for example-can also result in significant body weight reduction when combined with other lifestyle modifications. The aim is often a modest reduction in body weight of 5–10 %, which has indeed been demonstrated to enhance metabolic health and reduce the risk of obesity-related comorbidities [222].

The other component of behavioral interventions, and an essential one, is physical activity. According to recommendations from the U.S. Preventive Services Task Force, there should be at least 150 min of moderate-intensity or 75 min of vigorous-intensity aerobic physical activity per week, with strength training exercises included [223]. Regular physical activity not only provides weight loss but allows for cardiovascular improvement in this group of individuals and prohibits weight regain following weight loss [224].

Cognitive-behavioral therapy (CBT) has emerged as an effective approach to obesity management, as it identifies the psychosocial bases of unhealthy eating. CBT helps individuals recognize and modify maladaptive thoughts and behaviors related to food, stress, and body image [225]. The interventions in which CBT has been incorporated reportedly enhance emotional regulation, reduce binge eating, and enhance adherence to diet and exercise programs. This is especially important for ensuring long-term weight loss and avoiding recurrence [226].

**Fig. 7:**
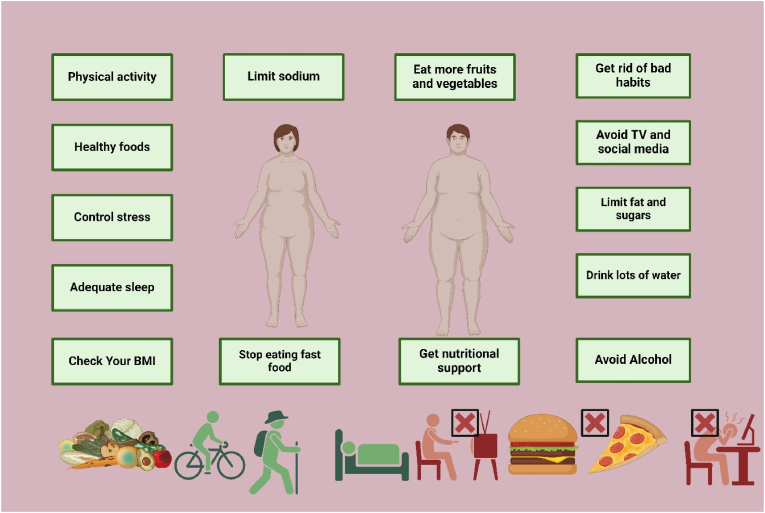
Various lifestyle modifications to prevent and manage obesity.

There is a growing incorporation of various forms of mindfulness-based approaches, including mindful eating, into behavioral treatments. These practices allow individuals to become more aware of internal signs of hunger and fullness to improve their self-regulation of eating and not engage in emotional eating or bingeing. The limited research conducted has indicated that the addition of mindfulness practices to dietary and physical activity interventions produces greater body mass index reductions and an overall improvement in eating behaviors [227]. Fig. 7 presents various lifestyle modifications to prevent and manage obesity, emphasizing the importance of physical activity, healthy eating habits, and stress control. Key strategies include reducing sodium and fast-food intake, consuming more fruits and vegetables, drinking plenty of water, and getting adequate sleep, alongside avoiding bad habits like excessive TV and social media use, limiting fat and sugar consumption, and seeking nutritional support (Fig. 7).

#### 1.7.2 Pharmacological interventions for management of obesity

Pharmacological interventions have become the critical component in treating obesity, particularly for those individuals who fail to achieve adequate weight loss by means of lifestyle modification. Such interventions are intended to facilitate weight loss by acting on appetite regulation, energy balance, and fat absorption. Several antiobesity drugs are approved for long-term treatment, with effectiveness and safety differing between them (Table 1).

**Table 1:** Obesity treatment drugs approved by the Food and Drug Administration (FDA).

Name	Mechanism of Action	Route of Administration	Recommended Dose	Expected Weight Loss, kg
Liraglutide	GLP-1 agonist	Subcutaneous	3 mg once a day	5.7–8
Semaglutide	GLP-1 agonist	Subcutaneous	2.4 mg once a week	9.7–15.3
Tirzepatide∗	GLP-1/GIP agonist	Subcutaneous	15 mg once a week	9.5–23.6
Orlistat	Lipase inhibitor	Oral	120 mg three times a day	5.8–10.6
Phentermine-Topiramate	Sympathomimetic amine anorectic/antiepileptic combination	Oral	7.5 mg/46 mg once a day	9.2–12.4
Bupropion-naltrexone	Opioid antagonist/antidepressant combination	Oral	16 mg/180 mg twice a day	3.6–9.3

Note: ∗Tirzepatide is still under evaluation by the FDA for obesity treatment approval.

Abbreviations: GLP-1, glucagon-like peptide-1; GIP, glucose-dependent insulinotropic polypeptide.

Notable examples of pharmacologic agents for the management of obesity include liraglutide and semaglutide, which are part of the GLP-1 receptor agonist class. Liraglutide has been shown to induce clinically significant reductions in body weight when administered at a dosage of 3.0 mg/day by inducing appetite-suppressing effects, thereby making the subject fuller. Semaglutide, given once weekly at 2.4 mg, has been shown to be more effective than other drugs available today [229]. It has been reported that these GLP-1 receptor agonists promote significant weight loss and, at the same time, improve other parameters of cardiometabolic outcomes, such as glycemic control and a reduction in blood pressure [230].

Other drugs include the drug combination of naltrexone/bupropion, which acts on the central nervous system to decrease appetite and food intake for pleasure [231]. Indeed, the combination has been good enough in inducing weight loss, mainly among those that cannot resist food out of emotional reasons or cravings. Nonetheless, this drug has commonly brought minimal rates of weight loss compared to GLP-1 receptor agonists; besides, it produces such side effects as nausea and headaches [232].

Orlistat-a lipase inhibitor that decreases the absorption of dietary fat-has been available for many years; however its use has also been limited due to gastrointestinal side effects, including oily stools and increased bowel movements [233]. Weight loss with orlistat is relatively more modest compared with newer therapies. Recent clinical guidelines suggest that orlistat should be considered only when other, more effective and better tolerated medications are not appropriate [228].

Among emerging pharmacological treatments, the dual agonist of GLP-1 and GIP receptors, tirzepatide, is promising for even greater weight loss than that achieved with current therapies [234]. These reflect the continuous evolution in obesity pharmacotherapy and allow hope for more effective treatments with good safety profiles in the future.

#### 1.7.3 Surgical interventions for management of obesity

Bariatric surgery is a well-established and highly effective treatment for severe obesity, particularly for patients who have failed to achieve substantial weight loss through either lifestyle modification or pharmacological interventions. It is associated with substantial long-term weight loss and improvement in comorbidities related to obesity, including type 2 diabetes, hypertension, and cardiovascular diseases. Recent literature has reassured the long-term merits of bariatric surgery in its capabilities to decrease all-cause mortality and prolong life expectancy, in obese patients with and without diabetes [235]. In one meta-analysis of over 174,772 subjects, noted were 49 % reductions in mortality from all causes after bariatric surgery, though life expectancy was extended by as high as 9.3 years in diabetic patients [236].

**Fig. 8:**
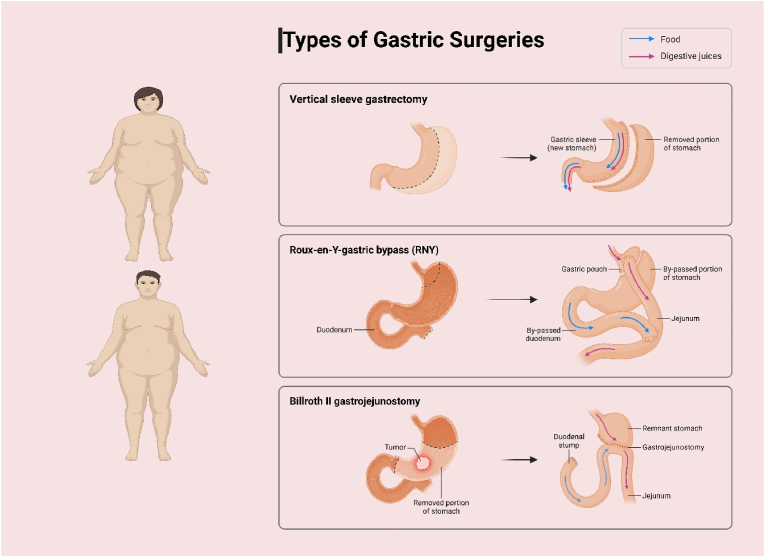
Illustrates three weight loss surgical interventions.

The two most common types of bariatric surgery, Roux-en-Y gastric bypass (RYGB) and sleeve gastrectomy (SG), result in similar weight loss patterns at five years, although the duodenal switch procedure tends to have even greater weight loss and a higher resolution of comorbidities. Most importantly, bariatric surgery was found to achieve remission of type 2 diabetes in a lot of patients, largely due to improvements in insulin sensitivity and changes in gut hormones after the procedure. Besides the effect of weight loss, there is a reduction in cardiovascular risk factors such as hypertension and dyslipidemia following bariatric surgery, which reduces the risk of long-term mortality. [237]. Fig. 8 illustrates three weight loss surgical interventions: Vertical Sleeve Gastrectomy, Roux-en-Y Gastric Bypass, and Billroth II Gastrojejunostomy. Each procedure affects the stomach and/or digestive tract differently to limit food intake or bypass parts of the gastrointestinal system in order to accomplish weight loss and mitigate other co-morbidities of obesity (Fig. 8).

Although bariatric surgery provides many benefits, the procedure is not without risks. Nutritional deficiencies following the course of changes in the absorptive capacity of nutrients after surgery are common complications, which are essential to maintain. Nutrient deficiencies and supplementation will require long-term follow-up care. Other potential long-term complications include weight regain and revisional surgery if suboptimal weight loss or other complications such as symptomatic GERD develop. Indeed, some studies have indicated that although RYGB offers superior outcomes in terms of long-term weight maintenance and resolution of GERD, SG has fewer short-term complications and may be preferred for selected patients [238].

In conclusion, bariatric surgery is a powerful intervention for severe obesity with impressive, long-term benefits related to weight loss, resolution of comorbidities, and overall mortality. It does, however, require careful selection of patients and lifelong follow-up for optimal outcomes and the management of potential risks.

#### 1.7.4 Lifestyle and environmental interventions

Community-based interventions have been one of the leading methods of tackling obesity in adults. Community-based programs can improve healthier behaviors through better access to more nutritious foods and increased opportunities for physical activity. The CDC has a high obesity program, which, in collaboration with land-grant universities, implements evidence-based policies and environmental changes with the goal of improving health that focuses on counties whose adult obesity rate exceeds 40 percent. The interventions aim to increase the availability of healthy foods and to promote physical activity through policy and environmental changes that support such behaviors. To date, more than 2 million people have been reached through the program-a demonstration of what can be achieved in large-scale, community-driven efforts for obesity [239].

Workplace wellness programs also play an important part in preventing obesity in adults. It targets employees through workplace wellness programs that provide resources regarding healthy food, exercise facilities, and education on health. Evidence shows that workplace interventions are effective in reducing body weight and improving general health, particularly when the focus of these interventions is nutrition and physical activity. Wellness programs may be particularly effective in male-dominated workplaces where it has been traditionally very difficult to engage employees in health promotion [240].

Public health campaigns can also be effective; these are usually supported by the government and local organizations in effecting healthy lifestyle changes. Examples include reducing sugar-sweetened drink consumption and increasing physical activity. They often take policy-driven approaches where there is a basis for implementing taxes on unhealthy foods or improving access to recreational facilities. Indeed, such a multifaceted intervention has been shown to create healthier behaviors and improve weight-related outcomes in large populations [241].

#### 1.7.5 Preventive strategies

Prevention strategies for obesity aim at early intervention and the sustenance of lifestyle modification in order to address the complex interaction of behavioral, environmental, and physiological components that are causative. Public health campaigns and policy-driven initiatives now meaningfully promote healthier environments. On the rise are the promulgation of public policies aimed at promoting physical activities, enhancing access to healthy foods, and placing restrictions on the marketing of unhealthy products. Indeed, studies have documented that population-level interventions, including the imposition of a tax on sweetened beverages and a clearer labeling of food items, result in modest yet significant reductions in dietary caloric intake and promote healthier eating behaviors [242].

Public health education has also played a very important role in the prevention of obesity, particularly by emphasizing early intervention. Awareness for healthy eating and physical activity raises the chances of behavior change regarding the prevention of conditions leading to obesity among populations at high risk. Early educational programs targeting adults and communities have been successful, especially those using new technologies and tailored approaches in changing knowledge and healthier lifestyles [243].

Lifestyle changes in a sustainable manner are crucial to preventing obesity. Lifestyle modification programs with intensive long-term changes in diet, exercise, and behavior are required for the maintenance of weight loss in the long term. Community or online support for long-term adherence is often emphasized besides individualized programs of dietary adjustment and increase in physical activities. Most effective interventions include behavioral counseling, which would help the individual with emotional eating by developing ways to cope with stress that would support long-term weight loss [222].

#### 1.7.6 Global policy interventions

Real-world policy interventions offer valuable insights into effective strategies for curbing obesity. Countries that have implemented bold regulatory and fiscal measures provide a template for evidence-based public health policy. In 2014, Mexico introduced a nationwide excise tax of 1 peso per liter (approximately 10 %) on sugar-sweetened beverages (SSBs), aiming to reduce consumption and tackle rising obesity and diabetes rates. Evaluations showed a 5.5 % reduction in SSB purchases in the first year, rising to 9.7 % in the second year, with greater declines among lower-income households [244]. Preliminary data also indicated increased water consumption as a healthier substitute. The tax has been lauded as a cost-effective measure to reduce obesity-related disease burdens and has inspired similar policies in other Latin American countries [245,246]. Japan implemented the “Metabo Law” in 2008, which mandates annual waist circumference screenings for adults aged 40–74 as part of the national metabolic syndrome prevention program. Individuals exceeding waist thresholds (≥85 cm for men and ≥90 cm for women) receive counseling and follow-up care [[247], [248], [249]]. The program emphasizes early detection and lifestyle modification, and has been associated with a stabilization of obesity rates and increased public awareness of health risks associated with abdominal obesity. Japan's approach reflects a culturally adapted, preventive model grounded in public health responsibility and corporate cooperation. In 2016, Chile introduced one of the world's most comprehensive food labeling laws, requiring front-of-package warning labels on high-sugar, high-fat, or high-salt foods. It also restricted marketing to children and banned the sale of such products in schools. Within 18 months, studies found a decrease in purchases of SSBs and a reduction in the marketing of unhealthy foods during children's programming [250]. The law has become a benchmark for policy innovation, demonstrating how structural interventions can shift consumer behavior and food industry practices. The UK's voluntary sugar reduction program launched in 2016, targeting a 20 % sugar reduction in foods commonly consumed by children. Although progress was mixed—with only a 3 % reduction achieved in most categories by 2020—the program succeeded in reducing sugar levels in soft drinks by over 40 % following the introduction of a soft drinks industry levy in 2018 [251]. These results suggest that voluntary measures may be less effective without accompanying fiscal or regulatory enforcement. These policy case studies highlight the importance of combining fiscal incentives, regulatory frameworks, public education, and environmental changes to address obesity at a population level. Tailoring strategies to local cultural, economic, and political contexts is essential for success and sustainability.

## 2 Implications for public health policy

The rising tide of obesity carries profound implications for health systems and policy-makers. Effective management of this epidemic demands a multi-sectoral public health response. Government policy intervention is vital to stem the obesity crisis, both to prevent new cases and to manage existing ones. Without decisive action, many countries risk their healthcare services becoming overwhelmed by obesity-related illnesses. The findings of this review support policies that create healthier food and physical activity environments – for example, implementing taxes on sugar-sweetened beverages, mandating clearer nutritional labeling, and restricting the marketing of unhealthy foods. Such population-level measures have been shown to yield modest but significant improvements in dietary habits and caloric intake. It is also crucial to address socioeconomic and regional disparities in obesity prevalence. High-income countries have plateaued at elevated obesity rates, while low- and middle-income countries are experiencing rapid increases, often alongside persistent undernutrition. This dual burden means policies must be tailored to local contexts – strengthening food security and nutrition on one hand, and curbing obesogenic factors on the other. In summary, public health policy should prioritize obesity prevention as a key component of non-communicable disease control, allocate resources for obesity treatment programs, and foster environments that make healthy choices easier for the population.

## 3 Future research directions

Despite extensive research, significant knowledge gaps remain in our understanding and treatment of obesity. Future research should continue to unravel the complex biological and social determinants of obesity – for instance, investigating gene–environment interactions, the gut microbiome's role in weight regulation, and the impact of early-life nutrition on long-term obesity risk. There is also a need for long-term studies on the **e**ffectiveness and sustainability of interventions. Many current treatments achieve only partial or temporary success, and issues like weight regain (notably after interventions such as diet programs or bariatric surgery) warrant further investigation. Emerging therapeutic approaches (for example, novel pharmacotherapies and hormone-based treatments) and supportive strategies (such as mindfulness-based therapies or digital health tools) show promise, but require more rigorous evaluation to establish their long-term efficacy and safety. Additionally, policy-focused research is crucial – scholars should examine the population-level impact of interventions like soda taxes, urban planning for active living, and school-based nutrition programs to guide evidence-based public health decisions. By addressing these research needs, the scientific community can provide deeper insights and innovative solutions, informing more effective strategies to combat obesity in the future.

## 4 Conclusion

Obesity is a multifaceted and escalating global health crisis that requires urgent, comprehensive intervention. This article highlights the complex interplay of genetic, environmental, behavioral, and socioeconomic factors contributing to the rising prevalence of obesity, which now affects over one billion individuals worldwide. The severe health consequences associated with obesity—including cardiovascular diseases, type 2 diabetes, certain cancers, and diminished quality of life—underscore its classification as a chronic disease requiring sustained management rather than short-term weight loss efforts. Effective public health policies must address obesogenic environments through regulatory measures such as food taxation, improved urban planning, and restrictions on unhealthy food marketing, while clinical management should integrate lifestyle modifications, pharmacotherapy, and, in severe cases, bariatric surgery. Future research should focus on refining long-term obesity interventions, exploring emerging therapeutic approaches, and evaluating policy-driven strategies to create sustainable, evidence-based solutions. Ultimately, reducing the global burden of obesity demands coordinated efforts at individual, community, and systemic levels to promote healthier lifestyles, equitable healthcare access, and robust preventive frameworks.

## 5 Recommendations for obesity prevention and management

Based on the findings of this article, several key recommendations can be made to guide practitioners and policy-makers in combating the obesity epidemic.

(1). **Adopt multi-sector preventive policies:** Implement comprehensive public health policies to shape healthier environments. Examples include fiscal measures (such as taxes on sugary drinks), regulations on food labeling and advertising, and urban planning that promotes physical activity. Such policies help reduce population-level risk factors and make healthy choices more accessible to all.
(2). **Prioritize lifestyle intervention programs:** Ensure that lifestyle modification remains the cornerstone of obesity management. Widespread programs should support individuals in improving diet quality and increasing physical activity, as these behavioral changes can yield a 5–10 % reduction in body weight, which significantly improves metabolic health and reduces obesity-related risks. Investing in community-based initiatives and counseling (nutrition education, exercise programs, behavioral therapy) is essential for both prevention and weight management.
(3). **Integrate clinical treatment options:** For individuals with established obesity, especially those with comorbid conditions, incorporate evidence-based clinical interventions. Pharmacotherapy (e.g. GLP-1 receptor agonists and other approved anti-obesity medications) can be offered as an adjunct to lifestyle changes for appropriate patients, with medical supervision. In cases of severe obesity, bariatric surgery should be considered as it can produce substantial and sustained weight loss and improve or resolve comorbidities. However, careful patient selection, multidisciplinary pre-operative evaluation, and lifelong post-surgical follow-up are recommended to optimize outcomes and manage risks.
(4). **Strengthen early intervention and education:** Emphasize early-life and lifelong obesity prevention through education and routine healthcare. This includes nutritional and physical education in schools, early screening for overweight in primary care, and family-based interventions to instill healthy habits from a young age. Public health campaigns should continue to raise awareness about the dangers of obesity and encourage lifestyle changes in all age groups. Health systems must also be prepared to treat obesity as a chronic condition, providing long-term support and follow-up for weight maintenance rather than just short-term weight reduction.

## CRediT authorship contribution statement

**Sirwan Khalid Ahmed:** Writing – review & editing, Writing – original draft, Resources, Project administration, Data curation, Conceptualization. **Ribwar Arsalan Mohammed:** Writing – review & editing, Writing – original draft, Supervision, Data curation, Conceptualization.

## Ethical approval

The ethical approval was not required, as the study conducted did not involve any ethical concerns or issues.

## Funding

No funds, grants, or other support were received in relation to this paper.

## Declaration of competing interests

The authors declare that they have no known competing financial interests or personal relationships that could have appeared to influence the work reported in this paper.

## References

[1] World Health Organization (2024). https://www.who.int/news/item/01-03-2024-one-in-eight-people-are-now-living-with-obesity#:%7E:text=Newstudyreleasedbythe,adultswereoverweightin2022.

[2] Westbury S., Oyebode O., van Rens T., Barber T.M. (2023). Obesity stigma: causes, consequences, and potential solutions. Curr Obes Rep.

[3] Chang Chusan Y.A., Eneli I., Hennessy E., Pronk N.P., Economos C.D. (2025). Next steps in efforts to address the obesity epidemic. Annu Rev Publ Health.

[4] Roomy M Al, Hussain K., Behbehani H.M., Abu-Farha J., Al-Harris R., Ambi A.M. (2024). Therapeutic advances in obesity management: an overview of the therapeutic interventions. Front Endocrinol.

[5] (2025). Global, regional, and national prevalence of child and adolescent overweight and obesity, 1990-2021, with forecasts to 2050: a forecasting study for the Global Burden of Disease Study 2021. Lancet (London, England).

[6] Collaborators G. (2025). Global, regional, and national prevalence of adult overweight and obesity, 1990-2021, with forecasts to 2050: a forecasting study for the Global Burden of Disease Study 2021. Lancet (London, England).

[7] Bray G.A. (2025). Obesity: a 100 year perspective. Int J Obes.

[8] Ng M., Fleming T., Robinson M., Thomson B., Graetz N., Margono C. (2014). Global, regional, and national prevalence of overweight and obesity in children and adults during 1980-2013: a systematic analysis for the Global Burden of Disease Study 2013. Lancet (London, England).

[9] Ford N.D., Patel S.A., Narayan K.M.V. (2017). Obesity in low- and middle-income countries: burden, drivers, and emerging challenges. Annu Rev Publ Health.

[10] Boutari C., Mantzoros C.S. (2022). A 2022 update on the epidemiology of obesity and a call to action: as its twin COVID-19 pandemic appears to be receding, the obesity and dysmetabolism pandemic continues to rage on. Metabolism.

[11] (2024). Worldwide trends in underweight and obesity from 1990 to 2022: a pooled analysis of 3663 population-representative studies with 222 million children, adolescents, and adults. Lancet (London, England).

[12] Gao L., Bhurtyal A., Wei J., Akhtar P., Wang L., Wang Y. (2020). Double burden of malnutrition and nutrition transition in Asia: a case study of 4 selected countries with different socioeconomic development. Adv Nutr.

[13] Nyanhanda T., Mwanri L., Mude W. (2023). Double burden of malnutrition: a population level comparative cross-sectional study across three sub-saharan African countries-Malawi, Namibia and Zimbabwe. Int J Environ Res Publ Health.

[14] Winichagoon P., Margetts B.M. (2017). The double burden of malnutrition in low-and middle-income countries. Energy Balanc Obes.

[15] Kroker-Lobos M.F., Pedroza-Tobías A., Pedraza L.S., Rivera J.A. (2014). The double burden of undernutrition and excess body weight in Mexico. Am J Clin Nutr.

[16] Tan P.Y., Chan C.L., Som S.V., Dye L., Moore J.B., Caton S. (2024). Prevalence and key determinants of the triple burden of childhood malnutrition in Southeast Asian countries: a systematic review and meta-analysis within an adapted socio-ecological framework. Crit Rev Food Sci Nutr.

[17] Pomati M., Mendoza-Quispe D., Anza-Ramirez C., Hernández-Vásquez A., Carrillo Larco R.M., Fernandez G. (2021). Trends and patterns of the double burden of malnutrition (DBM) in Peru: a pooled analysis of 129,159 mother–child dyads. Int J Obes.

[18] Pradeilles R., Landais E., Pareja R., Eymard‐Duvernay S., Markey O., Holdsworth M. (2023). Exploring the magnitude and drivers of the double burden of malnutrition at maternal and dyad levels in peri‐urban Peru: a cross‐sectional study of low‐income mothers, infants and young children. Matern Child Nutr.

[19] Lai W.K., Palaniveloo L., Mohd Sallehuddin S., Ganapathy S.S. (2024). Double burden of malnutrition and its socio-demographic determinants among children and adolescents in Malaysia: national Health and Morbidity Survey 2019. J Health Popul Nutr.

[20] Viana R.S., De Araújo-Moura K., De Moraes A.C.F. (2025). Worldwide prevalence of the double burden of malnutrition in children and adolescents at the individual level: systematic review and meta-regression. J Pediatr.

[21] Azomahou T.T., Diene B., Gosselin-Pali A. (2022). Transition and persistence in the double burden of malnutrition and overweight or obesity: evidence from South Africa. Food Policy.

[22] Kiosia A., Dagbasi A., Berkley J.A., Wilding J.P.H., Prendergast A.J., Li J.V. (2024). The double burden of malnutrition in individuals: Identifying key challenges and re‐thinking research focus. Nutr Bull.

[23] Talukder A., Kelly M., Gray D., Sarma H. (2024). Prevalence and trends of double burden of malnutrition at household-level in South and Southeast Asia. Discov Public Heal.

[24] Pencil A., Matsungo T.M., Chuchu T.M., Hongu N., Hayami N. (2024). The double burden of malnutrition among adolescents from Zimbabwe: a cross-sectional study. Obesities.

[25] Barazzoni R., Gortan Cappellari G. (2020). Double burden of malnutrition in persons with obesity. Rev Endocr Metab Disord.

[26] Viana R.S., Nascimento-Ferreira M.V., Schaan B.D., Bloch K.V., de Carvalho K.M.B., Cureau F.V. (2024). Prevalence of the double burden of malnutrition among adolescents: associations with lifestyle behaviors and Clusters of social determinants. Children.

[27] Mekonnen S., Birhanu D., Menber Y., Gebreegziabher Z.A., Belay M.A. (2024). Double burden of malnutrition and associated factors among mother–child pairs at household level in Bahir Dar City, Northwest Ethiopia: community based cross-sectional study design. Front Nutr.

[28] Carvajal-Aldaz D., Cucalon G., Ordonez C. (2022). Food insecurity as a risk factor for obesity: a review. Front Nutr.

[29] Morales M.E., Berkowitz S.A. (2016). The relationship between food insecurity, dietary patterns, and obesity. Curr Nutr Rep.

[30] Niedermayer F., Wolf K., Zhang S., Dallavalle M., Nikolaou N., Schwettmann L. (2024). Sex-specific associations of environmental exposures with prevalent diabetes and obesity – results from the KORA Fit study. Environ Res.

[31] Bellisario V., Comoretto R.I., Berchialla P., Koumantakis E., Squillacioti G., Borraccino A. (2022). The association between Greenness and urbanization level with weight status among adolescents: new evidence from the HBSC 2018 Italian survey. Int J Environ Res Publ Health.

[32] Anza-Ramirez C., Lazo M., Zafra-Tanaka J.H., Avila-Palencia I., Bilal U., Hernández-Vásquez A. (2022). The urban built environment and adult BMI, obesity, and diabetes in Latin American cities. Nat Commun.

[33] Guo Y., Yin X., Sun Y., Zhang T., Li M., Zhang F. (2022). Research on environmental influencing factors of overweight and obesity in children and adolescents in China. Nutrients.

[34] Moradi S., Mirzababaei A., Dadfarma A., Rezaei S., Mohammadi H., Jannat B. (2019). Food insecurity and adult weight abnormality risk: a systematic review and meta-analysis. Eur J Nutr.

[35] Brown A.G.M., Esposito L.E., Fisher R.A., Nicastro H.L., Tabor D.C., Walker J.R. (2019). Food insecurity and obesity: research gaps, opportunities, and challenges. Transl Behav Med.

[36] Farrell P., Thow A.M., Abimbola S., Faruqui N., Negin J. (2018). How food insecurity could lead to obesity in LMICs: when not enough is too much: a realist review of how food insecurity could lead to obesity in low- and middle-income countries. Health Promot Int.

[37] Agyemang C., Kushitor S.B., Afrifa-Anane G.F., de-Graft Aikins A., Ahima R.S. (2023). Obesity in Africa: a Silent public health crisis BT - metabolic syndrome: a comprehensive Textbook.

[38] Hossain M.B., Khan J.R., Adhikary A.C., Anwar A.H.M.M., Raheem E., Siddiqee M.H. (2022). Association between childhood overweight/obesity and urbanization in developing countries: evidence from Bangladesh. J Public Health.

[39] Du W., Wang H., Su C., Jia X., Zhang B. (2022). Thirty-year urbanization trajectories and obesity in Modernizing China. Int J Environ Res Publ Health.

[40] Kirchengast S., Hagmann D. (2021). “Obesity in the City” – urbanization, health risks and rising obesity rates from the viewpoint of human biology and public health. Hum Biol Public Heal.

[41] Mehata S., Shrestha N., Ghimire S., Atkins E., Karki D.K., Mishra S.R. (2021). Association of altitude and urbanization with hypertension and obesity: analysis of the Nepal Demographic and Health Survey 2016. Int Health.

[42] Das B., Khaled K.L. (2024). Pathophysiology of obesity: an extensive review. Indo Global J Pharmaceut Sci.

[43] Magkos F., Sørensen T.I.A., Raubenheimer D., Dhurandhar N.V., Loos R.J.F., Bosy-Westphal A. (2024). On the pathogenesis of obesity: causal models and missing pieces of the puzzle. Nat Metab.

[44] Rydin A.A., Severn C., Pyle L., Morelli N., Shoemaker A.H., Chung S.T. (2024). Prediction of resting energy expenditure for adolescents with severe obesity: a multi-centre analysis. Pediatr Obes.

[45] Johnson R.J., Sánchez-Lozada L.G., Lanaspa M.A. (2024). The fructose survival hypothesis as a mechanism for unifying the various obesity hypotheses. Obesity.

[46] Minari T.P., Manzano C.F., Yugar L.B.T., Sedenho-Prado L.G., de Azevedo Rubio T., Tácito L.H.B. (2024). Demystifying obesity: understanding, prevention, treatment, and stigmas. Nutr Rev.

[47] Manore M.M., Larson-Meyer D.E., Lindsay A.R., Hongu N., Houtkooper L. (2017). Dynamic energy balance: an integrated framework for discussing diet and physical activity in obesity prevention—is it more than eating less and exercising more?. Nutrients.

[48] Westerterp K.R. (2018). Exercise, energy balance and body composition. Eur J Clin Nutr.

[49] Hall K.D., Farooqi I.S., Friedman J.M., Klein S., Loos R.J.F., Mangelsdorf D.J. (2022). The energy balance model of obesity: beyond calories in, calories out. Am J Clin Nutr.

[50] Torres-Carot V., Suárez-González A., Lobato-Foulques C. (2022). The energy balance hypothesis of obesity: do the laws of thermodynamics explain excessive adiposity?. Eur J Clin Nutr.

[51] Polyzou E.A., Polyzos S.A. (2024). Outdoor environment and obesity: a review of current evidence. Metab Open.

[52] Jackson S.E., Llewellyn C.H., Smith L. (2020). The obesity epidemic - nature via nurture: a narrative review of high-income countries. SAGE Open Med.

[53] Gasmi A., Noor S., Menzel A., Pivina L., Bjørklund G. (2021). Obesity and insulin resistance: associations with chronic inflammation, genetic and epigenetic factors. Curr Med Chem.

[54] Lin X., Li H. (2021). Obesity: epidemiology, pathophysiology, and therapeutics. Front Endocrinol.

[55] Halder S.K., Melkani G.C. (2025). The interplay of genetic predisposition, circadian misalignment, and metabolic regulation in obesity. Curr Obes Rep.

[56] Autret K., Bekelman T.A. (2024). Socioeconomic status and obesity. J Endocr Soc.

[57] Hill D., Conner M., Clancy F., Moss R., Wilding S., Bristow M. (2022). Stress and eating behaviours in healthy adults: a systematic review and meta-analysis. Health Psychol Rev.

[58] Lopes Cortes M., Andrade Louzado J., Galvão Oliveira M., Moraes Bezerra V., Mistro S., Souto Medeiros D. (2021). Unhealthy food and psychological stress: the association between ultra-processed food consumption and perceived stress in working-class young adults. Int J Environ Res Publ Health.

[59] Spinosa J., Christiansen P., Dickson J.M., Lorenzetti V., Hardman C.A. (2019). From socioeconomic disadvantage to obesity: the mediating role of psychological distress and emotional eating. Obesity.

[60] Dakanalis A., Mentzelou M., Papadopoulou S.K., Papandreou D., Spanoudaki M., Vasios G.K. (2023). The association of emotional eating with overweight/obesity, depression, anxiety/stress, and dietary patterns: a review of the current clinical evidence. Nutrients.

[61] Welsh A., Hammad M., Piña I.L., Kulinski J. (2024). Obesity and cardiovascular health. Eur J Prev Cardiol.

[62] Powell-Wiley T.M., Poirier P., Burke L.E., Després J.-P., Gordon-Larsen P., Lavie C.J. (2021). Obesity and cardiovascular disease: a scientific statement from the American heart association. Circulation.

[63] Akil L., Ahmad H.A. (2011). Relationships between obesity and cardiovascular diseases in four southern states and Colorado. J Health Care Poor Underserved.

[64] Shah N.M., Kaltsakas G. (2023). Respiratory complications of obesity: from early changes to respiratory failure. Breathe.

[65] Liu C., Chen M.-S., Yu H. (2017). The relationship between obstructive sleep apnea and obesity hypoventilation syndrome: a systematic review and meta-analysis. Oncotarget.

[66] Orozco González B.N., Rodriguez Plascencia N., Palma Zapata J.A., Llamas Domínguez A.E., Rodríguez González J.S., Diaz J.M. (2024). Obesity hypoventilation syndrome, literature review. SLEEP Adv.

[67] Collins K.H., Herzog W., MacDonald G.Z., Reimer R.A., Rios J.L., Smith I.C. (2018). Obesity, metabolic syndrome, and musculoskeletal disease: common inflammatory pathways suggest a central role for loss of muscle integrity. Front Physiol.

[68] Nedunchezhiyan U., Varughese I., Sun A.R., Wu X., Crawford R., Prasadam I. (2022). Obesity, inflammation, and immune system in osteoarthritis. Front Immunol.

[69] Mocanu V., Timofte D.V., Zară-Dănceanu C.-M., Labusca L. (2024). Obesity, metabolic syndrome, and osteoarthritis require integrative understanding and management. Biomedicines.

[70] Li Z., Yin S., Zhao G., Cao X. (2024). Association between sarcopenic obesity and osteoarthritis: the potential mediating role of insulin resistance. Exp Gerontol.

[71] Henriques J., Berenbaum F., Mobasheri A. (2024). Obesity-induced fibrosis in osteoarthritis: pathogenesis, consequences and novel therapeutic opportunities. Osteoarthr Cartil Open.

[72] De Pergola G., Silvestris F. (2013). Obesity as a major risk factor for cancer. J Obes.

[73] Pati S., Irfan W., Jameel A., Ahmed S., Shahid R.K. (2023). Obesity and cancer: a current overview of epidemiology, pathogenesis, outcomes, and management. Cancers (Basel).

[74] Anastasiou I.A., Kounatidis D., Vallianou N.G., Skourtis A., Dimitriou K., Tzivaki I. (2025). Beneath the surface: the emerging role of ultra-processed foods in obesity-related cancer. Curr Oncol Rep.

[75] Stone T.W., McPherson M., Gail Darlington L. (2018). Obesity and cancer: existing and new hypotheses for a causal connection. EBioMedicine.

[76] Ramos-Nino M.E. (2013). The role of chronic inflammation in obesity-associated cancers. ISRN Oncol.

[77] World Health Organization Obesity. WHO 2024. https://www.who.int/health-topics/obesity#tab=tab_1.

[78] Mahase E. (2023). Global cost of overweight and obesity will hit $4.32tn a year by 2035, report warns. BMJ.

[79] world obesity (2024). Economic impact of overweight and obesity to surpass $4 trillion by 2035. World Obes.

[80] Puhl R.M., Himmelstein M.S., Pearl R.L. (2020). Weight stigma as a psychosocial contributor to obesity. Am Psychol.

[81] Ryan L., Coyne R., Heary C., Birney S., Crotty M., Dunne R. (2023). Weight stigma experienced by patients with obesity in healthcare settings: a qualitative evidence synthesis. Obes Rev.

[82] Alimoradi Z., Golboni F., Griffiths M.D., Broström A., Lin C.-Y., Pakpour A.H. (2020). Weight-related stigma and psychological distress: a systematic review and meta-analysis. Clin Nutr.

[83] Sikorski C., Spahlholz J., Hartlev M., Riedel-Heller S.G. (2016). Weight-based discrimination: an ubiquitary phenomenon?. Int J Obes.

[84] Hajek A., Kretzler B., König H.-H. (2021). The association between obesity and social isolation as well as Loneliness in the adult population: a systematic review. Diabetes Metab Syndr Obes.

[85] Wu Y.-K., Berry D.C. (2018). Impact of weight stigma on physiological and psychological health outcomes for overweight and obese adults: a systematic review. J Adv Nurs.

[86] World Health Organization (2024).

[87] World Health Organization (2024). https://www.who.int/news-room/fact-sheets/detail/obesity-and-overweight.

[88] Mehrzad R., Mehrzad R.B.T.-O. (2020). Obes Glob impact Epidemiol.

[89] Schumacher L.M., Ard J., Sarwer D.B. (2023). Promise and unrealized potential: 10 years of the American Medical Association classifying obesity as a disease. Front Public Health.

[90] Frühbeck G., Busetto L., Dicker D., Yumuk V., Goossens G.H., Hebebrand J. (2019). The ABCD of obesity: an EASO position statement on a diagnostic term with clinical and scientific implications. Obes Facts.

[91] Lingvay I., Cohen R.V., Roux CW le, Sumithran P. (2024). Obesity in adults. Lancet.

[92] World obesity atlas. World obesity atlas 2024: Obesiy and its consequences 2024. https://www.worldobesity.org/news/world-obesity-atlas-2024.

[93] El Bcheraoui C., Afshin A., Charara R., Khalil I., Moradi-Lakeh M., Kassebaum N.J. (2018). Burden of obesity in the eastern mediterranean region: findings from the global burden of disease 2015 study. Int J Publ Health.

[94] World Health Organization (WHO) (2025). https://www.who.int/news-room/fact-sheets/detail/obesity-and-overweight.

[95] Popkin B.M., Laar A. (2025). Nutrition transition's latest stage: are ultra-processed food increases in low- and middle-income countries dooming our preschoolers' diets and future health?. Pediatr Obes.

[96] Baker P., Machado P., Santos T., Sievert K., Backholer K., Hadjikakou M. (2020). Ultra‐processed foods and the nutrition transition: global, regional and national trends, food systems transformations and political economy drivers. Obes Rev.

[97] Popkin B.M., Ng S.W. (2022). The nutrition transition to a stage of high obesity and noncommunicable disease prevalence dominated by ultra-processed foods is not inevitable. Obes Rev an Off J Int Assoc Study Obes.

[98] Weihrauch-Blüher S., Schwarz P., Klusmann J.-H. (2019). Childhood obesity: increased risk for cardiometabolic disease and cancer in adulthood. Metabolism.

[99] Sahoo K., Sahoo B., Choudhury A.K., Sofi N.Y., Kumar R., Bhadoria A.S. (2015). Childhood obesity: causes and consequences. J Fam Med Prim Care.

[100] Singh A.S., Mulder C., Twisk J.W.R., Van Mechelen W., Chinapaw M.J.M. (2008). Tracking of childhood overweight into adulthood: a systematic review of the literature. Obes Rev.

[101] Simmonds M., Llewellyn A., Owen C.G., Woolacott N. (2016). Predicting adult obesity from childhood obesity: a systematic review and meta‐analysis. Obes Rev.

[102] Rundle A.G., Factor-Litvak P., Suglia S.F., Susser E.S., Kezios K.L., Lovasi G.S. (2020). Tracking of obesity in childhood into adulthood: effects on body mass index and fat mass index at age 50. Child Obes.

[103] Wang Y., Cai L., Wu Y., Wilson R.F., Weston C., Fawole O. (2015). What childhood obesity prevention programmes work? A systematic review and meta‐analysis. Obes Rev.

[104] World Health Organization (WHO) (2021). https://www.who.int/initiatives/making-every-school-a-health-promoting-school.

[105] Ramirez A., Fox K., Herrera Y.M., Gans K.M., Risica P.M., McCurdy K. (2024). Goals, barriers, and Facilitators of caregivers who participated in an in-home intervention to improve food Parenting practices and child diet quality. J Nutr Educ Behav.

[106] Perdew M., Liu S., Naylor P.-J. (2021). Family-based nutrition interventions for obesity prevention among school-aged children: a systematic review. Transl Behav Med.

[107] Enright G., Allman-Farinelli M., Redfern J. (2020). Effectiveness of family-based behavior change interventions on obesity-related behavior change in children: a realist synthesis. Int J Environ Res Publ Health.

[108] Fulkerson J.A., Friend S., Horning M., Flattum C., Draxten M., Neumark-Sztainer D. (2018). Family home food environment and nutrition-related parent and child personal and behavioral outcomes of the healthy home offerings via the mealtime environment (HOME) plus program: a randomized controlled trial. J Acad Nutr Diet.

[109] Black A.P., D'Onise K., McDermott R., Vally H., O'Dea K. (2017). How effective are family-based and institutional nutrition interventions in improving children's diet and health? A systematic review. BMC Public Health.

[110] Gans K.M., Tovar A., Kang A., Ward D.S., Stowers K.C., von Ash T. (2022). A multi-component tailored intervention in family childcare homes improves diet quality and sedentary behavior of preschool children compared to an attention control: results from the Healthy Start-Comienzos Sanos cluster randomized trial. Int J Behav Nutr Phys Activ.

[111] Golan M., Crow S. (2004). Parents are key players in the prevention and treatment of weight-related problems. Nutr Rev.

[112] Roberto C.A., Swinburn B., Hawkes C., Huang T.T.K., Costa S.A., Ashe M. (2015). Patchy progress on obesity prevention: emerging examples, entrenched barriers, and new thinking. Lancet.

[113] Wharton S., Lau D.C.W., Vallis M., Sharma A.M., Biertho L., Campbell-Scherer D. (2020). Obesity in adults: a clinical practice guideline. Can Med Assoc J.

[114] Piché M.-E., Tchernof A., Després J.-P. (2020). Obesity Phenotypes, diabetes, and cardiovascular diseases. Circ Res.

[115] Cesaro A., De Michele G., Fimiani F., Acerbo V., Scherillo G., Signore G. (2023). Visceral adipose tissue and residual cardiovascular risk: a pathological link and new therapeutic options. Front Cardiovasc Med.

[116] Wani K., Kumar B., Al-Daghri N.M., Sabico S. (2024). Trends and characteristics of the metabolically healthy obese phenotype in an Arab population. Front Public Health.

[117] Agius R., Pace N.P., Fava S. (2024). Phenotyping obesity: a focus on metabolically healthy obesity and metabolically unhealthy normal weight. Diabetes Metab Res Rev.

[118] Janota O., Mantovani M., Kwiendacz H., Irlik K., Bucci T., Lam S.H.M. (2024). Metabolically “extremely unhealthy” obese and non-obese people with diabetes and the risk of cardiovascular adverse events: the Silesia Diabetes-Heart Project. Cardiovasc Diabetol.

[119] Pluta W., Dudzińska W., Lubkowska A. (2022). Metabolic obesity in people with normal body weight (MONW)-Review of Diagnostic Criteria. Int J Environ Res Publ Health.

[120] Pledger S.L., Ahmadizar F. (2023). Gene-environment interactions and the effect on obesity risk in low and middle-income countries: a scoping review. Front Endocrinol.

[121] Huang C., Chen W., Wang X. (2023). Studies on the fat mass and obesity-associated (FTO) gene and its impact on obesity-associated diseases. Genes Dis.

[122] Gross D.C., Cheever C.R., Batsis J.A. (2023). Understanding the development of sarcopenic obesity. Expet Rev Endocrinol Metabol.

[123] Jung H.N., Jung C.H., Hwang Y.-C. (2023). Sarcopenia in youth. Metabolism.

[124] Park H.-K., Ahima R.S. (2023). Endocrine disorders associated with obesity. Best Pract Res Clin Obstet Gynaecol.

[125] Meligi AAH El, Ahmed R.M., Shaltout I., Soliman A.R. (2024). Exploring obesity-related endocrine disorders beyond diabetes: a narrative review. Egypt J Intern Med.

[126] Wu Y., Li D., Vermund S.H. (2024). Advantages and limitations of the body mass index (BMI) to assess adult obesity. Int J Environ Res Publ Health.

[127] Jeong S.-M., Lee D.H., Rezende L.F.M., Giovannucci E.L. (2023). Different correlation of body mass index with body fatness and obesity-related biomarker according to age, sex and race-ethnicity. Sci Rep.

[128] Kesztyüs D., Lampl J., Kesztyüs T. (2021). The weight problem: overview of the most common concepts for body mass and fat distribution and critical consideration of their Usefulness for risk assessment and practice. Int J Environ Res Publ Health.

[129] Chait A., Den Hartigh L.J. (2020). Adipose tissue distribution, inflammation and its metabolic consequences, including diabetes and cardiovascular disease. Front Cardiovasc Med.

[130] Ross R., Neeland I.J., Yamashita S., Shai I., Seidell J., Magni P. (2020). Waist circumference as a vital sign in clinical practice: a consensus Statement from the IAS and ICCR working group on visceral obesity. Nat Rev Endocrinol.

[131] Carvalho V.C.H.D.S., Moreira L.B., Luft V.C., Fuchs S.C. (2023). Waist-to-Height ratio: a sensitive tool for assessing the need for nutritional risk management in Elderly populations from Brazil. Healthc (Basel, Switzerland).

[132] World Health Organization (WHO) Waist circumference and waist-hip ratio: report of a WHO expert consultation n.d. https://www.who.int/publications/i/item/9789241501491.

[133] Yoo E.-G. (2016). Waist-to-height ratio as a screening tool for obesity and cardiometabolic risk. Korean J Pediatr.

[134] Savva S.C., Lamnisos D., Kafatos A.G. (2013). Predicting cardiometabolic risk: waist-to-height ratio or BMI. A meta-analysis. Diabetes, Metab Syndr Obes Targets Ther.

[135] Aguirre C., Tumani M.F., Carrasco F., Inostroza J., Obregón A.M., Reyes Á. (2024). Relative fat mass as an estimator of body fat percentage in Chilean adults. Eur J Clin Nutr.

[136] Woolcott O.O., Bergman R.N. (2020). Defining cutoffs to diagnose obesity using the relative fat mass (RFM): association with mortality in NHANES 1999–2014. Int J Obes.

[137] Silva A.M., Campa F., Stagi S., Gobbo L.A., Buffa R., Toselli S. (2023). The bioelectrical impedance analysis (BIA) international database: aims, scope, and call for data. Eur J Clin Nutr.

[138] Monda A., de Stefano M.I., Villano I., Allocca S., Casillo M., Messina A. (2024). Ultra-processed food intake and increased risk of obesity: a narrative review. Foods.

[139] Concepción-Zavaleta M.J., Quiroz-Aldave J.E., Durand-Vásquez M del C., Gamarra-Osorio E.R., Valencia de la Cruz J., del C., Barrueto-Callirgos C.M. (2024). A comprehensive review of genetic causes of obesity. World J Pediatr.

[140] Loos R.J.F., Yeo G.S.H. (2022). The genetics of obesity: from discovery to biology. Nat Rev Genet.

[141] Melhorn S.J., Askren M.K., Chung W.K., Kratz M., Bosch T.A., Tyagi V. (2018). FTO genotype impacts food intake and corticolimbic activation. Am J Clin Nutr.

[142] Fan S., Chen S., Lin L. (2023). Research progress of gut microbiota and obesity caused by high-fat diet. Front Cell Infect Microbiol.

[143] Lee C.J., Sears C.L., Maruthur N. (2020). Gut microbiome and its role in obesity and insulin resistance. Ann N Y Acad Sci.

[144] Engin A. (2024). The mechanism of leptin resistance in obesity and therapeutic perspective. Obes Lipotoxicity.

[145] Anderson K.C., Hasan F., Grammer E.E., Kranz S. (2023). Endogenous ghrelin levels and perception of hunger: a systematic review and meta-analysis. Adv Nutr.

[146] Noor J., Chaudhry A., Batool S., Noor R., Fatima G. (2023). Exploring the impact of the gut microbiome on obesity and weight loss: a review article. Cureus.

[147] Magne F., Gotteland M., Gauthier L., Zazueta A., Pesoa S., Navarrete P. (2020). The Firmicutes/Bacteroidetes ratio: a Relevant marker of gut Dysbiosis in obese patients?. Nutrients.

[148] Zhang K., Zhang Q., Qiu H., Ma Y., Hou N., Zhang J. (2024). The complex link between the gut microbiome and obesity-associated metabolic disorders: mechanisms and therapeutic opportunities. Heliyon.

[149] Benbaibeche H., Saidi H., Bounihi A., Koceir E.A. (2023). Emotional and external eating styles associated with obesity. J Eat Disord.

[150] Moschonis G., Trakman G.L. (2023). Overweight and obesity: the interplay of eating habits and physical activity. Nutrients.

[151] Woessner M.N., Tacey A., Levinger-Limor A., Parker A.G., Levinger P., Levinger I. (2021). The evolution of technology and physical inactivity: the good, the bad, and the way forward. Front Public Health.

[152] Thivel D., Tremblay M.S., Chaput J.-P. (2013). Modern sedentary behaviors favor energy consumption in children and adolescents. Curr Obes Rep.

[153] Al-Jawaldeh A., Abbass M.M.S. (2022). Unhealthy dietary habits and obesity: the major risk factors beyond non-communicable diseases in the eastern mediterranean region. Front Nutr.

[154] Mititelu M., Oancea C.-N., Neacşu S.M., Musuc A.M., Gheonea T.C., Stanciu T.I. (2023). Evaluation of Junk food consumption and the risk related to consumer health among the Romanian population. Nutrients.

[155] Smith J., Ang X.Q., Giles E.L., Traviss-Turner G. (2023). Emotional eating interventions for adults living with overweight or obesity: a systematic review and meta-analysis. Int J Environ Res Publ Health.

[156] Scott K.A., Melhorn S.J., Sakai R.R. (2012). Effects of chronic social stress on obesity. Curr Obes Rep.

[157] Goens D., Virzi N.E., Jung S.E., Rutledge T.R., Zarrinpar A. (2023). Obesity, chronic stress, and stress reduction. Gastroenterol Clin N Am.

[158] Howell N.A., Booth G.L. (2022). The weight of place: built environment Correlates of obesity and diabetes. Endocr Rev.

[159] Jiang Y., Wang S., Ren L., Yang L., Lu Y. (2022). Effects of built environment factors on obesity risk across three types of residential community in Beijing. J Transport Health.

[160] Lam T.M., Vaartjes I., Grobbee D.E., Karssenberg D., Lakerveld J. (2021). Associations between the built environment and obesity: an umbrella review. Int J Health Geogr.

[161] García de Jalón S., Chiabai A., Quiroga S., Suárez C., Ščasný M., Máca V. (2021). The influence of urban greenspaces on people's physical activity: a population-based study in Spain. Landsc Urban Plann.

[162] Cereijo L., Gullón P., Del Cura I., Valadés D., Bilal U., Badland H. (2022). Exercise facilities and the prevalence of obesity and type 2 diabetes in the city of Madrid. Diabetologia.

[163] Anderson E., Wei R., Liu B., Plummer R., Kelahan H., Tamez M. (2021). Improving healthy food choices in low-income settings in the United States using behavioral economic-based adaptations to choice Architecture. Front Nutr.

[164] Selvakumaran S., Lin C.-Y., Hadgraft N., Chandrabose M., Owen N., Sugiyama T. (2023). Area-level socioeconomic inequalities in overweight and obesity: systematic review on moderation by built-environment attributes. Health Place.

[165] Griffith R. (2022). Obesity, poverty and public policy. Econ J.

[166] Hemmingsson E. (2014). A new model of the role of psychological and emotional distress in promoting obesity: conceptual review with implications for treatment and prevention. Obes Rev.

[167] Lee K.M., Wang C., Du H., Hunger J., Tomiyama A.J. (2024). Weight stigma as a stressor: a preliminary multi-wave, longitudinal study testing the biobehavioral pathways of the cyclic obesity/weight-based stigma (COBWEBS) model. Appetite.

[168] Zhu H., Zhao K., Huang L., Shi W., Tang C., Xu T. (2024). Individual, family and social-related factors of eating behavior among Chinese children with overweight or obesity from the perspective of family system. Front Pediatr.

[169] Uzun M.E., Kara Ö., Şirin H., Kaymaz N. (2023). Examination of relationship factors between psychological resilience and social support in adolescent obesity. Arch Pediatr.

[170] Biondi B. (2024). Subclinical hypothyroidism in patients with obesity and metabolic syndrome: a narrative review. Nutrients.

[171] Mateo R.C.I., Hennessey J.V. (2019). Thyroxine and treatment of hypothyroidism: seven decades of experience. Endocrine.

[172] Bavaresco A., Mazzeo P., Lazzara M., Barbot M. (2024). Adipose tissue in cortisol excess: what Cushing's syndrome can teach us?. Biochem Pharmacol.

[173] Frias-Toral E., Garcia-Velasquez E., de Los Angeles Carignano M., Rodriguez-Veintimilla D., Alvarado-Aguilera I., Bautista-Litardo N. (2021). Polycystic ovary syndrome and obesity: clinical aspects and nutritional management. Minerva Endocrinol.

[174] Mazereel V., Detraux J., Vancampfort D., Van Winkel R., De Hert M. (2020). Impact of psychotropic medication effects on obesity and the metabolic syndrome in people with serious mental illness. Front Endocrinol.

[175] Moitra S., Carsin A.-E., Abramson M.J., Accordini S., Amaral A.F.S., Anto J. (2023). Long-term effect of asthma on the development of obesity among adults: an international cohort study, ECRHS. Thorax.

[176] Musaiger A.O. (2011). Overweight and obesity in eastern mediterranean region: prevalence and possible causes. J Obes.

[177] Oguoma V.M., Coffee N.T., Alsharrah S., Abu-Farha M., Al-Refaei F.H., Al-Mulla F. (2021). Prevalence of overweight and obesity, and associations with socio-demographic factors in Kuwait. BMC Public Health.

[178] Weiderpass E., Botteri E., Longenecker J.C., Alkandari A., Al-Wotayan R., Al Duwairi Q. (2019). The prevalence of overweight and obesity in an adult Kuwaiti population in 2014. Front Endocrinol.

[179] Salem V., AlHusseini N., Abdul Razack H.I., Naoum A., Sims O.T., Alqahtani S.A. (2022). Prevalence, risk factors, and interventions for obesity in Saudi Arabia: a systematic review. Obes Rev.

[180] Jaacks L.M., Slining M.M., Popkin B.M. (2015). Recent underweight and overweight trends by rural–urban residence among women in low-and middle-income Countries1, 2. J Nutr.

[181] Kruger H.S., Puoane T., Senekal M., Van Der Merwe M.-T. (2005). Obesity in South Africa: challenges for government and health professionals. Public Health Nutr.

[182] Akpan E.E., Ekpenyong C.E. (2013). Urbanization Drift and obesity epidemic in sub-Saharan Africa: a review of the Situation in Nigeria. Eur J Sustain Dev.

[183] Popkin B.M., Corvalan C., Grummer-Strawn L.M. (2020). Dynamics of the double burden of malnutrition and the changing nutrition reality. Lancet.

[184] Kroll F., Swart E.C., Annan R.A., Thow A.M., Neves D., Apprey C. (2019). Mapping obesogenic food environments in South Africa and Ghana: correlations and contradictions. Sustainability.

[185] Wang W., Mensah I.A., Atingabili S., Omari-Sasu A.Y., Nouwati E., Kunkuaboor C.Y. (2025). Obesity Kuznets Curve conjecture assessment in African economies: conditioning effects of urbanization, food, and trade using gender-based regional analysis. Glob Health.

[186] Drewnowski A. (2009). Obesity, diets, and social inequalities. Nutr Rev.

[187] Galic S., Oakhill J.S., Steinberg G.R. (2010). Adipose tissue as an endocrine organ. Mol Cell Endocrinol.

[188] Myers M.G., Leibel R.L., Seeley R.J., Schwartz M.W. (2010). Obesity and leptin resistance: distinguishing cause from effect. Trends Endocrinol Metabol.

[189] Lumeng C.N., Saltiel A.R. (2011). Inflammatory links between obesity and metabolic disease. J Clin Investig.

[190] Houstis N., Rosen E.D., Lander E.S. (2006). Reactive oxygen species have a causal role in multiple forms of insulin resistance. Nature.

[191] Ritov V.B., Menshikova E.V., He J., Ferrell R.E., Goodpaster B.H., Kelley D.E. (2005). Deficiency of subsarcolemmal mitochondria in obesity and type 2 diabetes. Diabetes.

[192] Milagro F.I., Mansego M.L., De Miguel C., Martínez J.A. (2013). Dietary factors, epigenetic modifications and obesity outcomes: progresses and perspectives. Mol Aspect Med.

[193] Godfrey K.M., Costello P.M., Lillycrop K.A. (2015). The developmental environment, epigenetic biomarkers and long-term health. J Dev Orig Health Dis.

[194] Turnbaugh P.J., Ley R.E., Mahowald M.A., Magrini V., Mardis E.R., Gordon J.I. (2006). An obesity-associated gut microbiome with increased capacity for energy harvest. Nature.

[195] Cani P.D., Amar J., Iglesias M.A., Poggi M., Knauf C., Bastelica D. (2007). Metabolic endotoxemia initiates obesity and insulin resistance. Diabetes.

[196] Pakhare M., Anjankar A. (2024). Critical correlation between obesity and cardiovascular diseases and recent Advancements in obesity. Cureus.

[197] Kalil G.Z., Haynes W.G. (2012). Sympathetic nervous system in obesity-related hypertension: mechanisms and clinical implications. Hypertens Res.

[198] Ebong I.A., Goff D.C., Rodriguez C.J., Chen H., Bertoni A.G. (2014). Mechanisms of heart failure in obesity. Obes Res Clin Pract.

[199] Shiozawa M., Kaneko H., Itoh H., Morita K., Okada A., Matsuoka S. (2021). Association of body mass index with ischemic and hemorrhagic stroke. Nutrients.

[200] Chandrasekaran P., Weiskirchen R. (2024). The role of obesity in type 2 diabetes Mellitus—an overview. Int J Mol Sci.

[201] Jha B.K., Sherpa M.L., Imran M., Mohammed Y., Jha L.A., Paudel K.R. (2023). Progress in understanding metabolic syndrome and knowledge of its complex pathophysiology. Diabetology.

[202] Bays H.E., Kirkpatrick C., Maki K.C., Toth P.P., Morgan R.T., Tondt J. (2024). Obesity, dyslipidemia, and cardiovascular disease: a joint expert review from the obesity medicine association and the national lipid association 2024. Obes Pillars.

[203] Yki-Järvinen H. (2014). Non-alcoholic fatty liver disease as a cause and a consequence of metabolic syndrome. Lancet Diabetes Endocrinol.

[204] Nousseir H.M. (2019). Obesity: the major preventable risk factor of obstructive sleep apnea. J Curr Med Res Pract.

[205] Seetho I.W., Wilding J.P.H., Sbraccia P., Finer N. (2018). Obesity and obstructive sleep apnea syndrome BT - obesity: pathogenesis, diagnosis, and treatment.

[206] Masa J.F., Pépin J.-L., Borel J.-C., Mokhlesi B., Murphy P.B., Sánchez-Quiroga M.Á. (2019). Obesity hypoventilation syndrome. Eur Respir Rev.

[207] Tashiro H., Kurihara Y., Kuwahara Y., Takahashi K. (2024). Impact of obesity in asthma: possible future therapies. Allergol Int.

[208] Chen N., Fong D.Y.T., Wong J.Y.H. (2023). Health and economic outcomes associated with musculoskeletal disorders attributable to high body mass index in 192 countries and Territories in 2019. JAMA Netw Open.

[209] Terkawi M.A., Ebata T., Yokota S., Takahashi D., Endo T., Matsumae G. (2022). Low-grade inflammation in the pathogenesis of osteoarthritis: cellular and molecular mechanisms and strategies for future therapeutic intervention. Biomedicines.

[210] Lucha-López M.O., Hidalgo-García C., Monti-Ballano S., Márquez-Gonzalvo S., Ferrández-Laliena L., Müller-Thyssen-Uriarte J. (2023). Body mass index and its influence on chronic low back pain in the Spanish population: a secondary analysis from the European health survey (2020). Biomedicines.

[211] Cannata F., Vadalà G., Ambrosio L., Fallucca S., Napoli N., Papalia R. (2020). Intervertebral disc degeneration: a focus on obesity and type 2 diabetes. Diabetes Metab Res Rev.

[212] Mao T., He Q., Yang J., Jia L., Xu G. (2024). Relationship between gout, hyperuricemia, and obesity—does central obesity play a significant role?—a study based on the NHANES database. Diabetol Metab Syndr.

[213] Marin A.-G., Filipescu A., Petca A. (2024). The role of obesity in the Etiology and carcinogenesis of endometrial cancer. Cureus.

[214] Harvey S.V., Wentzensen N., Bertrand K., Black A., Brinton L.A., Chen C. (2023). Associations of life course obesity with endometrial cancer in the Epidemiology of Endometrial Cancer Consortium (E2C2). Int J Epidemiol.

[215] Gonzalez-Gutierrez L., Motiño O., Barriuso D., de la Puente-Aldea J., Alvarez-Frutos L., Kroemer G. (2024). Obesity-associated colorectal cancer. Int J Mol Sci.

[216] Gluba-Brzózka A., Rysz J., Ławiński J., Franczyk B. (2022). Renal cell cancer and obesity. Int J Mol Sci.

[217] Parra-Landazury N.M., Cordova-Gallardo J., Méndez-Sánchez N. (2021). Obesity and gallstones. Visc Med.

[218] Alexandre L., Long E., Beales I.L. (2014). Pathophysiological mechanisms linking obesity and esophageal adenocarcinoma. World J Gastrointest Pathophysiol.

[219] Chen Y., Wang W., Morgan M.P., Robson T., Annett S. (2023). Obesity, non-alcoholic fatty liver disease and hepatocellular carcinoma: current status and therapeutic targets. Front Endocrinol.

[220] Nagle C.M., Dixon S.C., Jensen A., Kjaer S.K., Modugno F., deFazio A. (2015). Obesity and survival among women with ovarian cancer: results from the Ovarian Cancer Association Consortium. Br J Cancer.

[221] Gostoli S., Raimondi G., Popa A.P., Giovannini M., Benasi G., Rafanelli C. (2024). Behavioral lifestyle interventions for weight loss in overweight or obese patients with type 2 diabetes: a systematic review of the literature. Curr Obes Rep.

[222] Wadden T.A., Tronieri J.S., Butryn M.L. (2020). Lifestyle modification approaches for the treatment of obesity in adults. Am Psychol.

[223] Bull F.C., Al-Ansari S.S., Biddle S., Borodulin K., Buman M.P., Cardon G. (2020). World Health Organization 2020 guidelines on physical activity and sedentary behaviour. Br J Sports Med.

[224] Swift D.L., McGee J.E., Earnest C.P., Carlisle E., Nygard M., Johannsen N.M. (2018). The effects of exercise and physical activity on weight loss and maintenance. Prog Cardiovasc Dis.

[225] Castelnuovo G., Pietrabissa G., Manzoni G.M., Cattivelli R., Rossi A., Novelli M. (2017). Cognitive behavioral therapy to aid weight loss in obese patients: current perspectives. Psychol Res Behav Manag.

[226] Moraes A. dos S., Padovani R. da C., La Scala Teixeira C.V., Cuesta M.G.S., Gil S. dos S., de Paula B. (2021). Cognitive behavioral approach to treat obesity: a randomized clinical trial. Front Nutr.

[227] Spadaro K.C., Davis K.K., Sereika S.M., Gibbs B.B., Jakicic J.M., Cohen S.M. (2018). Effect of mindfulness meditation on short-term weight loss and eating behaviors in overweight and obese adults: a randomized controlled trial. J Compl Integr Med.

[228] Telci Caklili O., Cesur M., Mikhailidis D.P., Rizzo M. (2023). Novel anti-obesity therapies and their different effects and safety profiles: a critical overview. Diabetes, Metab Syndr Obes.

[229] Rubino D.M., Greenway F.L., Khalid U., O'Neil P.M., Rosenstock J., Sørrig R. (2022). Effect of weekly subcutaneous semaglutide vs daily liraglutide on body weight in adults with overweight or obesity without diabetes: the STEP 8 randomized clinical trial. JAMA.

[230] Grunvald E., Shah R., Hernaez R., Chandar A.K., Pickett-Blakely O., Teigen L.M. (2022). AGA clinical practice guideline on pharmacological interventions for adults with obesity. Gastroenterology.

[231] Moawad M.H., Sadeq M.A., Abbas A., Ghorab R.M., Serag I., Hendawy M. (2024). Efficacy of naltrexone/bupropion in treatment of binge eating: a systematic review and meta-analysis. Psychiatry Int.

[232] Barrea L., Pugliese G., Muscogiuri G., Laudisio D., Colao A., Savastano S. (2020). New-generation anti-obesity drugs: naltrexone/bupropion and liraglutide. An update for endocrinologists and nutritionists. Minerva Endocrinol.

[233] Aydin B., Onbasi K. (2021). Lipase inhibitor orlistat: an old but still effective weapon. Med Sci.

[234] Son J.W. (2022). Recent advances in anti-obesity drugs. J Korean Diabetes.

[235] Carlsson L.M.S., Carlsson B., Jacobson P., Karlsson C., Andersson-Assarsson J.C., Kristensson F.M. (2023). Life expectancy after bariatric surgery or usual care in patients with or without baseline type 2 diabetes in Swedish Obese Subjects. Int J Obes.

[236] Syn N.L., Cummings D.E., Wang L.Z., Lin D.J., Zhao J.J., Loh M. (2021). Association of metabolic–bariatric surgery with long-term survival in adults with and without diabetes: a one-stage meta-analysis of matched cohort and prospective controlled studies with 174 772 participants. Lancet.

[237] Roth A.E., Thornley C.J., Blackstone R.P. (2020). Outcomes in bariatric and metabolic surgery: an updated 5-year review. Curr Obes Rep.

[238] Kamocka A., Parmar C., Kurzatkowski K., Chidambaram S., Goh E.L., Erridge S. (2021). Outcomes of bariatric surgery in extreme obesity: results from the United Kingdom National Bariatric Surgery Registry for patients with a body mass index >70 kg/m2. Surg Obes Relat Dis.

[239] Murriel A.L., Kahin S., Pejavara A., O'Toole T. (2020). The high obesity program: overview of the centers for disease control and prevention and cooperative extension services efforts to address obesity. Prev Chronic Dis.

[240] Bezzina A., Ashton L., Watson T., James C.L. (2022). Workplace wellness programs targeting weight outcomes in men: a scoping review. Obes Rev.

[241] Holston D., Stroope J., Cater M., Kendall M., Broyles S. (2020). Implementing policy, systems, and environmental change through community coalitions and extension partnerships to address obesity in rural Louisiana. Prev Chronic Dis.

[242] Souza L.M.S., Chaves S.C.L., Santana J.M., Pereira M. (2023). Public policy interventions for preventing and treating obesity: scoping review. Nutr Rev.

[243] Navidad L., Padial-Ruz R., González M.C. (2021). Nutrition, physical activity, and new technology programs on obesity prevention in primary education: a systematic review. Int J Environ Res Publ Health.

[244] Colchero M.A., Rivera-Dommarco J., Popkin B.M., Ng S.W. (2017). In Mexico, evidence of sustained consumer response two years after implementing a sugar-sweetened beverage tax. Health Aff.

[245] Basto-Abreu A., Torres-Alvarez R., Barrientos-Gutierrez T., Pereda P., Duran A.C. (2024). Estimated reduction in obesity prevalence and costs of a 20% and 30% ad valorem excise tax to sugar-sweetened beverages in Brazil: a modeling study. PLoS Med.

[246] Thow A.M., Downs S.M., Mayes C., Trevena H., Waqanivalu T., Cawley J. (2018). Fiscal policy to improve diets and prevent noncommunicable diseases: from recommendations to action. Bull World Health Organ.

[247] Ikeda N., Takimoto H., Imai S., Miyachi M., Nishi N. (2015). Data resource profile: the Japan National Health and nutrition survey (NHNS). Int J Epidemiol.

[248] Manzenreiter W. (2012). Monitoring health and the body: anthropometry, lifestyle risks, and the Japanese obesity crisis. J Jpn Stud.

[249] Lange K.W., Nakamura Y. (2024). Japanese food culture and human health–what we can learn from Japan. J Dis Prev Heal Promot.

[250] Taillie L.S., Reyes M., Colchero M.A., Popkin B., Corvalán C. (2020). An evaluation of Chile's Law of Food Labeling and Advertising on sugar-sweetened beverage purchases from 2015 to 2017: a before-and-after study. PLoS Med.

[251] gov.uk Sugar reduction: progress report, 2015 to 2019. GovUk 2020. https://www.gov.uk/government/publications/sugar-reduction-report-on-progress-between-2015-and-2019.

